# Socioeconomic position and access to supportive cancer care in Europe: a scoping review of literature from 2000 to 2024

**DOI:** 10.1007/s11764-025-01904-3

**Published:** 2025-10-07

**Authors:** Esther C. E. de Jongh, Gunn Ammitzbøll, Lonneke V. van de Poll-Franse, Eva Boomstra, Stevie van der Mierden, Susanne O. Dalton, Martijn M. Stuiver

**Affiliations:** 1 Department of Psychosocial Research and Epidemiology, Netherlands Cancer Institute, Amsterdam, The Netherlands; 2 Danish Cancer Society Research Center, Cancer Survivorship, Copenhagen, Denmark; 3 Danish Research Center for Equality in Cancer (COMPAS), Department of Clinical Oncology & Palliative Care, Zealand University Hospital, Naestved, Denmark; 4 Tilburg School of Social and Behavioral Sciences, Tilburg University, Tilburg, The Netherlands; 5 Scientific Information Service, Netherlands Cancer Institute, Amsterdam, The Netherlands; 6 Department of Clinical Medicine, Copenhagen University, Copenhagen, Denmark; 7 Faculty of Health, Amsterdam University of Applied Sciences, Amsterdam, The Netherlands

**Keywords:** Supportive care, Europe, Health disparities, Socioeconomic position, Psychosocial services, Cancer rehabilitation

## Abstract

**Purpose:**

Cancer prevalence is steadily rising in Europe. Many survivors experience symptoms and late effects that require management by supportive care. The inequities related to socioeconomic position (SEP) that have been described for primary anti-cancer treatment are likely to extend to the supportive care domain. This scoping review provides an overview of the existing literature assessing this relationship, and the methods used to do so.

**Methods:**

A comprehensive search was executed in May 2024 in multiple databases. Studies published after 2000, including real-world data on access to, uptake of, or adherence to supportive care in relation to SEP were included.

**Results:**

A total of 29 articles were included for review. Studies reported on a variety of supportive care types and were conducted primarily in Northern and Western Europe. Most studies with statistically significant findings (*n* = 14) reported an association between high SEP and increased access to or uptake of supportive care (*n* = 12).

**Conclusions:**

Research on the association of SEP with access and uptake of supportive care is increasing but still limited. Methodology varies and is mostly exploratory. The majority of the included studies indicate that socioeconomic differences in engagement with supportive cancer care in Europe persist. More research is needed to explain the causal pathways through which SEP affects engagement with supportive care, and how its accessibility can be improved in different European countries.

**Implications for Cancer Survivors:**

Efforts should be made across healthcare systems to improve accessibility to supportive cancer care for patients with a low SEP.

**Supplementary Information:**

The online version contains supplementary material available at https://doi.org/10.1007/s11764-025-01904-3.

## Introduction

Access to and uptake of general cancer treatment are not evenly distributed among those affected by the disease, as patients with lower socioeconomic position (SEP) face more barriers in accessing different treatment options [1, 2]. This is reflected in the poor cancer outcomes and decreased quality of life individuals in lower SEP experience compared to their more privileged counterparts [2–6]. In oncology, supportive care entails a range of services designed to improve patient well-being throughout the cancer care continuum. These include pain management, physical rehabilitation, dietary advice, and psychosocial, spiritual or social support [7]. Such forms of care have the potential to alleviate the negative consequences of anticancer treatment faced by cancer patients [8–10]. However, disparities in access to and uptake of supportive care may be particularly pronounced, as it is often not considered an integral part of standard anti-cancer treatment [11–13].

There are many possible explanations for why the supportive care needs of patients are unmet or fulfilled differently. Factors regarding individual capacity, such as lower health literacy, financial insecurity, or negative attitudes towards the healthcare system can influence patients’ receptiveness to additional care [6, 14–19]. Additionally, low SEP is negatively associated with an individual’s ability to self-determine, and the subsequent ability to be an active agent in their own disease trajectory, which can limit engagement in medical encounters – an important route through which supportive care needs are often identified or discussed [19–23].

Communication and engagement may also be affected. Patients with a lower SEP tend to participate less in medical consultations with healthcare professionals (HCPs) compared to their higher SEP counterparts [19, 20].

At the same time, system-level influences play a role; HCPs are central to making supportive care available, and although healthcare systems assume objectivity and prioritize vulnerable patients, this process can be affected by implicit assumptions or prioritization of more immediate treatment needs [23–29].

Compared to access to anti-cancer treatment and survival outcomes in relation to SEP, the area of socioeconomic differences in access to or uptake of supportive care in cancer is still underexplored [1, 2, 16, 18, 30, 31]. Previous reviews show inequity in access to cancer care, yet these have been conducted overseas (such as the U.S.) or have a global scope, assess cancer specific populations, assess primary cancer care disparities, or assess supportive care needs of patients (and not whether they are met) [30–32]. Importantly, these findings cannot be applied to European context for several reasons [33, 34]. Although there is some variation among national healthcare systems within Europe, the overarching models of healthcare funding and infrastructure are generally comparable. They do, however, differ substantially from those in for example the United States [34]. Further, in the European context, migration status plays a role, but is not recorded consistently due to legal and ethical constraints [77, 81]. Hence, SEP is commonly determined by attained education, income or material deprivation [36–38].

This scoping review will document the existing literature on access to, uptake of and adherence to supportive cancer care in European countries, its relationship to SEP, and report on which methods have been used to document this relationship. This scoping review addresses an important knowledge gap and formulates directions for future research, while also informing policies aimed at reducing barriers to supportive care. In doing so, this review aims to contribute to improving equitable access to, uptake of and adherence to supportive care to all cancer patients in need in Europe.

## Methods

### Identification of studies

This scoping review was performed according to the PRISMA-ScR guidelines for reporting and Joanna Briggs Institute (JBI) methodology guidelines for scoping reviews [39, 40]. A protocol was developed and uploaded to the Open Science Framework (OSF) registry in May 2024 [41]. The search string was iteratively developed in consultation with the information specialist (S.M.). and all research team members. We limited the search to articles after 2000, to provide adequate reflection of the current state of research and practice in Europe. The final search was executed on May 7, 2024 through PubMed, CINAHL, EMBASE, and PsycINFO for eligible articles published between 2000 and 2024. We did not consider abstracts or grey literature. The complete search strategy is available in Appendix A.

### Steps for selection

**Fig. 1:**
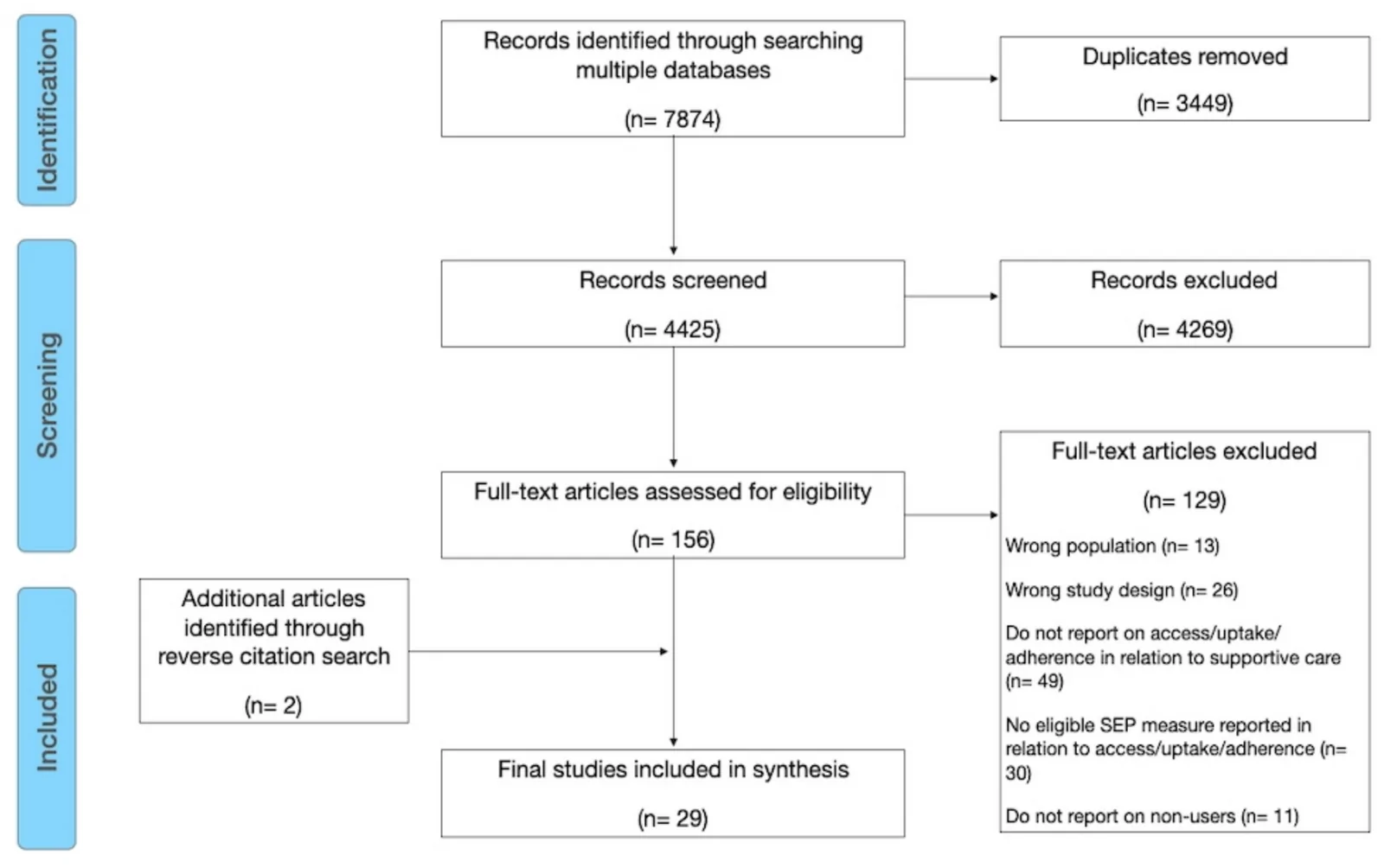
PRISMA flow diagram of the search process

After duplicates were removed, 4425 articles were imported into Rayyan [Rayyan Systems Inc, 2016] for the screening and selection process [42]. Prior to screening based on title and abstract, the literature reviewers (G.A., E.J.) each screened two consecutive samples mutually blinded, followed by a consensus session to resolve any conflicts. Two samples of 5% (*n* = 232) and 12% (*n* = 511) of the imported studies were screened and discussed to assure alignment on the in- and exclusion criteria and improve consensus over time. The initial absolute disagreement was 12% in the first sample and 5% in the second sample. Conflicts were considered on a case-by-case basis and unresolved cases were discussed among the broader research team if the literature reviewers did not reach agreement. In- and exclusion criteria were elaborated where necessary. After refinement and finalization of the eligibility criteria, the remainder of the articles were divided equally and screened by one of the reviewers based on title and abstract (G.A. or E.J.). Any articles marked as ‘maybe’ during this stage were discussed between both reviewers. A total of 178 articles were carried forward for full text screening and were independently considered by both reviewers. Any undecided articles (labelled ‘maybe’ by one of the reviewers) were discussed during consensus meetings to prevent potentially eligible articles from being misinterpreted or overlooked. The PRISMA flow diagram (Fig. 1) shows the stepwise search process, including the reasons for exclusion after full text review.

### Selection criteria

We included publications that were published in a peer-reviewed journal between 2000 and 2024. The population of interest included cancer patients in Europe, who were at least 18 years old and diagnosed with any cancer type. Supportive care was defined in alignment with the definition of the Multinational Association for Supportive Care in Cancer (MASCC) as “the prevention and management of the adverse effects of cancer and its treatment. This includes the management of physical and psychological symptoms and side effects across the continuum of the cancer journey from diagnosis through treatment to post-treatment care” [7]. We limited our search to supportive care received after cancer diagnosis and introduced by healthcare providers or supported in the clinical context. Information provided as supportive care or about supportive care, that was not considered standard anti-cancer treatment, was considered as well. We excluded complementary or alternative therapies that are not consistent with the conventional philosophical paradigm of western medicine (i.e., not based on our current scientific understanding of anatomy and physiology) and not supported by the health care setting. Studies on end-of-life care were also excluded.

Studies were considered if they included one or more of the predetermined objective SEP measures: education level, income level, or area or postal code. These variables provide insight into a person’s socioeconomic advantage or disadvantage by reflecting their access to social and economic resources. Composite measures of SEP that included more than the predefined measures without reporting on them separately were excluded, as this would have introduced ambiguity regarding which construct was driving the observed associations.

Any study design that presented real-world quantitative data on access, uptake, or adherence to supportive care was considered, if information on both users and non-users of supportive care was included. This included prospective and retrospective observational studies, registry-based analyses, and survey research, as long as empirical data on engagement with supportive care was included. *Access* was defined as receiving the opportunity to access supportive care; *uptake* was defined as actual utilization of the supportive care; and *adherence* was defined as continued or sustained use of supportive care over time. Randomized controlled trials (RCTs), feasibility studies, as well as studies assessing the need for supportive care were excluded.

Studies were retained for full text screening if they included the population of interest and reported on the predefined measures of SEP and their relation to supportive care. Individual studies were discussed among the entire research team if their eligibility remained unclear after full-text review.

### Data extraction

One researcher (E.J.) extracted relevant data from the included articles using a Microsoft Office Excel template that was developed in consultation with the research team members. Collected information included, but was not limited to, study design, authors, year of publication, country, population characteristics, type of supportive care, type of engagement, measure of SEP, outcome measures, data source, and information regarding statistical methods and analyses (e.g. adjustment, significance (as reported by the authors), findings). Accurate completion of the data extraction table was verified by a second researcher (G.A.).

## Results

### Study characteristics

The included studies are summarized in Table 1. A total of 29 studies were included for data synthesis, of which two studies were identified based on the reference lists of included articles after full text review. Sample sizes ranged from 168 to 19,865. The study designs included cross-sectional studies (*N* = 13), prospective cohort designs (*N* = 9) and historical cohort designs, either through prospective (*N* = 1) or retrospective data collection (*N* = 6). Most studies were published in the last decade (*n* = 23), and were conducted in Germany, Denmark, or the Netherlands (*n* = 22). All studies assessed education as a measure for SEP, and 9 studies additionally assessed income.

**Table 1:** Characteristics of the included studies

	Count (*n* = 29)
Year of publication	
Before 2015	6
2015–2019	10
2020–2024	13
Country*	
Germany	14
Denmark	4
The Netherlands	4
Sweden	2
Switzerland	2
France	1
Poland	1
UK	1
Spain	1
Supportive care type*	
Information about supportive care	1
Information as supportive care	1
Physical rehabilitation services, e.g., physiotherapy, treatment for erectile dysfunction, and speech therapy	7
Psychosocial services, e.g., psychological services, counseling, and social work	17
Rehabilitation services (according to patient needs or not specified)	8
Supportive care setting*	
Inpatient setting	13
Outpatient setting	4
Municipality-based rehabilitation services	3
According to patient needs or not specified	12
Type of engagement with care	
Access, i.e., given the opportunity for care (offer/referral/contact)	7
Uptake, i.e., engaging with, accepting care (use/participation/receive)	24
Socioeconomic position (SEP) measure*	
Education	29
Income	9
Sample size	
Under 500	11
500–1500	4
1500–3000	3
3000–5000	6
5000–10,000	2
10,000–20,000	3
Study design	
Cross-sectional study	13
Historical cohort study with prospective data collection	1
Historical cohort study with retrospective data collection	6
Prospective cohort study	9

*If an article reported on multiple categories it was counted in both categories

We included two sets of studies that drew from the same cohort. The Danish studies by Moustsen [43] and Dalton [44] used the same population-based registry but included patients from different, albeit overlapping, time periods; both were therefore included. Similarly, the two studies by Breidenbach [45, 46] used the same cohort but examined different types of supportive care. As these studies assess distinct aspects of the respective cohorts, they were considered unique and included in the synthesis.

### Categorization of studies

We categorized articles both according to *setting* in which the care was offered as well as *type of supportive care* (Table 1). Some studies only described in which setting the supportive care was organized (inpatient, outpatient, municipality based (i.e., offered in the primary healthcare sector)) [43, 44, 64–66], whereas other studies (only) specified the type of supportive care assessed. If a study described multiple types of care (e.g., psychosocial and physical), it was added to both categories.

### Types of supportive care

Seventeen studies reported on psychosocial services, of which 16 assessed the relationship with SEP as the primary outcome [45–59, 84, 87]. Studies that were categorized as ‘psychosocial services’ reported on psychological counseling [47], social work [45, 54], or a combination [50–52, 55, 59]. Seven studies reported on physical rehabilitation services as a primary outcome [48, 49, 56, 59–61, 87], and cohort designs were used to do so in 4 of them [56, 59, 61, 87]. Most of them reported on general physical rehabilitation initiatives (such as physiotherapy), only Stromberg et al. studied the effect of SEP on erectile dysfunction in prostate cancer specifically [61]. Two included studies reported on information *about* or information *as* supportive care using a cross-sectional design [62, 63].

### Access or uptake

The included studies only evaluated access or uptake, and none evaluated adherence to supportive care. Terminology regarding patient engagement with supportive care varied across studies; however, we categorized studies as ‘access’ to supportive care when terms like ‘offer,’ ‘referral,’ and ‘contact’ were used. Access to care was measured both through patient self-report [52, 54, 58, 62, 63], and obtained through registry data [43, 44, 61]. Studies describing engagement with care through terms like ‘use,’ ‘participate’, or ‘receive’ were categorized as assessing ‘uptake.’ Uptake of supportive care was measured through self-report by the majority of the studies [48–51, 53, 55–57, 59, 60, 64, 84–88], followed by the use of registry data [44–47, 65, 85]. One study measured uptake of supportive care through observing referral by the physician in agreement with the patient [66].

### Socioeconomic position (SEP)

Studies differed in how they measured and categorized income and education, ranging from self-report [57, 63] to registry data [44, 48, 59]. Income was categorized by quartiles or quintiles according to 5-year average, disposable income or income during time of data collection. For example, the study by Singer et al. calculated income based on self-reported household net income, divided by the number of individuals in the household [57]. The study by Holm et al. obtained income over a five-year period from national registries and calculated the average [48]. Likewise, education was categorized in different ways, i.e. according to length of education, level of degree, or completion of an education. The study by Orange et al. measured education through self-report and classified it according to nationally defined educational levels [63]. The study by Dalton et al. relied on national registries for both income and education, categorizing education by duration [44]. None of the included studies used area or postal code as a marker for SEP.

### Study characteristics and outcomes

Table 2 includes detailed information per study, including the aim, the study design, data source, and findings. Table 2 also includes whether multivariable adjustment was applied in the statistical analysis and if the adjustment set was explained where applicable. In doing so, we differentiated between studies explicitly aiming to estimate or test the total or direct causal effect of SEP, and those employing a more exploratory approach including SEP as one of several factors potentially contributing to the explained variance in uptake but not considered or treated as the central determinant. Some studies provided unadjusted intergroup comparison through counts of uptake, whereas others provided regression estimates (ORs) with confidence intervals (Appendix B). Two studies reported on hazard ratios (HRs) over time for attending rehabilitation services [40, 41].

**Table 2:** Complete overview of the included studies. Appendix B includes the table with estimates where applicable

Author Year Country	Relationship to supportive care type Purpose	Study design (*N*) Data source SEP measure Statistical method	Multivariable adjustment		Finding (between SEP and access/uptake) *Statistically significant indicated with an asterisk
			Education	Income	
Hensel, M 2002 Germany	Uptake of rehabilitation services (according to patient needs or not specified) *To determine if long-term QOL and re-employment following ASCT are related to sociodemographic factors (age, gender, educational level)*	Cross-sectional study (304) Questionnaire; clinical records Education Descriptive statistics	No adjustment		No association Educational level was the same in participants and non- participants of rehabilitation
Waldmann, A 2007 Germany	Uptake of rehabilitation services (according to patient needs or not specified) *To identify factors influencing rehabilitation utilization behavior*	Historical cohort study with retrospective data collection (241) POPGEN registry; questionnaire Education Multivariate analysis	Adjusted for personal, clinical and therapeutic variables		No significant influence of education on the use of rehabilitation services could be shown Not significant
Geyer, S 2012 Germany	Uptake of rehabilitation services (according to patient needs or not specified) *To investigate whether the utilization of inpatient oncological rehabilitation (within one year after surgery) shows social inequalities and which reasons lead to non-utilization*	Prospective cohort study (238) Interview data Education Logistic regression analysis	Adjusted for (at least): the number of children under 12 in the household, tumor stage, type of follow up treatment		The probability of using oncological rehabilitation decreased with increased education levels Not significant
Nicolaije, K.A.H 2012 The Netherlands	Access to information about supportive care *To identify the socio-demographic and clinical characteristics associated with information receipt*	Cross-sectional study (742) Questionnaire (EORTC-QLQ INFO25); Cohort registry (PROFILES) Education Multivariate linear and binary regression analysis	Adjusted for: sociodemographic and clinical characteristics (not further specified)		High SEP was associated with higher information receipt No statistically significant correlation found between education and perceived information provision on supportive care services (‘other services’)
Author Year Country	Relationship to supportive care type Purpose	Study design (*N*) Data source SEP measure Statistical method	Multivariable adjustment		Finding (between SEP and access/uptake) *Statistically significant indicated with an asterisk
			Education*	Income*	
von Heymann-Horan, A.B 2012 Denmark	Uptake of psychosocial services *To identify factors related to use of publicly subsidized psychotherapy, which were subsequently evaluated with regard to equity*	Prospective cohort study (3646) Questionnaire; national registry Education Multivariate cox regression models	Adjusted for: age at diagnosis, gender, education, previous psychotherapy use, emotional support from partner, children, and other family members, with SF-36 mental and physical component summaries, Charlson index scores		Longer education was associated with greater likelihood of initiating psychotherapy*
Holm, L.V 2013 Denmark	Uptake of psychosocial services; physical rehabilitation services *To analyze possible associations between cancer survivors’ SES and their participation in rehabilitation activities*	Population-based cohort study (3439) Questionnaire; National registry; Clinical records Education; income Univariate and multiple logistic regression analyses	Adjusted for: age group and cancer diagnosis		• Women with a higher education and to some extent higher income had increased odds of participating in activities* • Men with a higher income had increased odds of participating in physical activities* • A high education was associated with participation in psychological activities for both men and women*
Author Year Country	Relationship to Supportive care type Purpose	Study design (*n*) Data source SEP measure Statistical method	Multivariable adjustment		Finding (between SEP and access/uptake) *Statistically significant indicated with an asterisk
Zeissig, S.R 2015 Germany	Uptake of psychosocial services *To investigate how many cancer patients receive psycho-oncological care in hospital or in cancer counseling centers and which factors influence this utilization (e.g., education and income)*	Historical cohort study with retrospective data collection (6143) Questionnaire Education; income Multivariate logistic regression analysis	Adjusted for: gender, age, place of residence, education, income, and tumor spread at diagnosis		A higher level of education was significantly associated with the use of cancer counseling and the use of psycho-oncological services*, but income level was not
Ture, M 2015 Switzerland	Uptake of rehabilitation services (according to patient needs or not specified) *To evaluate whether there are differences in sociodemographic and medical characteristics between users and non-users of inpatient rehabilitation*	Cross-sectional study (238) Questionnaire; clinical records Education Univariate analysis	No multivariable adjustment	-	Stratified analyses showed that male patients with lower education were more likely to use rehabilitation*
Moustsen, I.R 2015 Denmark	Access to rehabilitation services (according to patient needs) *To examine the association between socioeconomic position and referral to rehabilitation services among cancer patients*	Population-based cohort study (13,840) National registry; Danish Cancer Registry Education; income Multivariate analysis	Adjusted for: sex, age, diagnosis, disposable income, cohabitation status and no. of children living at home		Higher educational level is associated with a higher probability of referral to rehabilitation services*
Arts, L.P.J 2018 The Netherlands	Uptake of psychosocial services *To identify sociodemographic and clinical factors associated with the use of psychosocial care services*	Cross-sectional study (1444) Questionnaire; Cohort registry (PROFILES) Education Multivariate logistic regression analysis	Adjusted for: age, sex comorbidities	-	Low education level was associated with increased use of psychosocial care services Non-significant
Author Year Country	Relationship to Supportive care type Purpose	Study design (*n*) Data source SEP measure Statistical method	Multivariable adjustment		Finding (between SEP and access/uptake) *Statistically significant indicated with an asterisk
			Education*	Income*	
Ernst, J 2018 Germany	Access to psychosocial services *To analyze the frequency and predictors of recommendations for psychosocial care and examine the use of these services by patients*	Cross-sectional study (4020) Questionnaire Education Binary regression analysis	Adjusted for: Sociodemographic, psychosocial, disease related factors	-	Higher education had a strong effect on recommendation/referral to psychosocial support services*
Garmy, P 2018 Sweden	Uptake of psychosocial services; physical rehabilitation services *To investigate the cancer rehabilitation experiences of working-age cancer survivors and explore the characteristics associated with these services*	Cross-sectional study (168) Questionnaire Education Descriptive statistics	No adjustment	-	No association
Hofreuter-Gätgens, K 2018 Germany	Uptake of psychosocial services; physical rehabilitation services *To examine social inequalities in the utilization of medical rehabilitation, (complementary and alternative medicine,) and psychosocial support programs among patients with prostate and breast cancer after surgery*	Prospective cohort study (455) Questionnaires Education; income Logistic regression analysis	Adjusted for: age, disease severity, psychological comorbidities		Only in breast cancer patients did education increase the likelihood of participating in psychosocial rehabilitation*
Weis, J 2018 Germany	Uptake of psychosocial services *To assess which factors are associated with the utilization of psychological care during inpatient care*	Cross-sectional study (1398) Questionnaire; clinical records Education Multivariate logistic regression analysis	Adjusted for: age, sex, cancer type	-	Higher education level was associated with the use of psychological care Non-significant
Author Year Country	Relationship to supportive care type Purpose	Study design (*N*) Data source SEP measure Statistical method	Multivariable adjustment		Finding (between SEP and access/uptake) *Statistically significant indicated with an asterisk
			Education*	Income*	
Dalton, S.O 2019 Denmark	Access to; uptake of rehabilitation services (according to patient needs) *To investigate associations between indicators for socioeconomic position and referral and attendance to rehabilitation for cancer*	Population-based cohort study (13,059) National registry; Danish cancer registry Education; income Multivariate cox regression models	Stratified by: sex, age, year of diagnosis Adjusted for: Disposable income, cohabitation, cancer type	Stratified by: sex, age, year of diagnosis Adjusted for: cohabitation, cancer type, highest attained education	Compared to individuals with short education, men and women with long education and men with medium education had higher adjusted hazard ratios (HR) for being referred to rehabilitation (*access*)*. Long education was associated with attendance (*uptake*) in both genders while in men*, medium education and in women high income, respectively was associated with attendance (*uptake*)*
Schuurhuizen, C.S.E.W 2019 The Netherlands	Uptake of psychosocial services *To identify predictors for use of psychosocial services in patients with metastatic colorectal cancer (mCRC) receiving first line chemotherapy enrolled in a prospective cluster randomized trial (CRT)*	Secondary analysis of trial data (349) Data from TES trial (NTR4034) Education Two-step multivariate analysis	Adjusted for: need to talk to professional, pain score at baseline, age, education (included in the multivariable model)	-	In this sample of patients with mCRC patients who completed higher education levels were found to be more likely to use psychosocial services.*
Breidenbach, C 2020 Germany	Uptake of psychosocial services *To Identify patients and center characteristics that elucidate the use of psycho-oncological services (POS) by patients in prostate cancer centers*	Prospective cohort study (3094) Questionnaire; TrueNTH Global registry Education Multilevel analysis	Adjusted for: individual patient and structural center characteristics	-	No association
Osowiecka, K 2020 Poland	Uptake of psychosocial services *To determine non-medical and organizational needs among cancer patients during diagnosis and treatment*	Cross-sectional study (384) (Orally administered) questionnaire Education Descriptive statistics; Chi-square test	No information provided		No association
Author Year Country	Relationship to Supportive care type Purpose	Study design (*N*) Data source SEP measure Statistical method	Multivariable adjustment		Finding (between SEP and access/uptake) *Statistically significant indicated with an asterisk
			Education*	Income*	
Ousseine, Y.M 2020 France	Access to psychosocial services *To study medico-social follow-up and its associated determinants with a focus on health literacy 5 years after diagnosis*	Cross-sectional study (4045) (Orally administered) questionnaire; FNHI registry Education; income Multivariate analysis	Adjusted for: sociodemographic, medical, and psychosocial characteristics		Contact with a social worker increased with a low income*
Breidenbach, C 2021 Germany Switzerland	Uptake of psychosocial services *To investigate patient and center-level predictors that explain variations in social service counseling utilization between centers*	Prospective cohort study (19,865) Questionnaire; TrueNTH Global Registry Education Logistic multilevel analysis	Adjusted for: sociodemographic and clinical patient information, therapy information	-	Patients with an entrance certificate for university have a lower likelihood to use social service counseling than patients with lower secondary school education
Noeres, D 2021 Germany	Uptake of rehabilitation services (according to patient needs) *To investigate predictors of participation in oncological rehabilitation within one year after primary breast cancer surgery*	Prospective cohort study (409) Questionnaires Education Multivariate analysis	Adjusted for: physical and mental health scores (SF-12)		Higher education had a significant effect on the likelihood of participating in rehabilitation*
Orange, S.T 2021 UK	Access to information as supportive care *To explore experiences of receiving physical activity advice post-diagnosis and determine the influence of sociodemographic characteristics on the recall of physical activity advice*	Cross-sectional study (242) Questionnaire Education Univariate generalized linear model	Adjustment for confounders: demographic characteristics	-	There was no evidence of associations between sociodemographic characteristics and the recall of physical activity advice
Author Year Country	Relationship to supportive care type Purpose	Study design (*N*) Data source SEP measure Statistical method	Multivariable adjustment		Finding (between SEP and access/uptake) *Statistically significant indicated with an asterisk
			Education*	Income*	
Scherer-Trame, S 2021 Germany	Uptake of rehabilitation services (according to patient needs or not specified) *To assess use of inpatient rehabilitation therapy (IRT), determinants of utilization and the association between IRT and survival in colorectal cancer patients*	Retrospective cohort study (3704) DACHS Study data; National registry; Clinical records Education Multivariate logistic regression analysis	Adjustment for confounders: age, sex, partnership, education, employment status, type of health insurance, BMI, smoking, physical activity level, cancer stage, tumor location, Charlson Comorbidity Score, chemotherapy, radio- therapy, surgical technique and surgical volume of treating hospital		There were no significant (inverse) associations between years of school education and IRT use
Korn, P 2022 Germany	Uptake of psychosocial services; physical rehabilitation services *To evaluate whether socioeconomic aspects affect oral cancer patients’ participation in rehabilitation treatment*	Retrospective cohort study (1532) Questionnaire; Cohort registry (DÖSAK) Education Intergroup comparison	No information provided	-	Education levels did not seem to be decisive in determining rehabilitation participation, difference was not statistically significant
Author Year Country	Relationship to supportive care type Purpose of study	Study design (*n*) Data source SEP measure Statistical method	Multivariable adjustment		Finding (between SEP and access/uptake) *Statistically significant indicated with an asterisk
			Education*	Income*	
Singer, S 2022 Germany	Uptake of; access to psychosocial services *To examine the pattern of psychosocial care in breast cancer survivors*	Prospective cohort study (456) Questionnaire; clinical records Education; income	Adjusted for: all other variables in the model (age, migration status, risk, financial issues)		Education and income were not statistically significant predictors of offer or use for both social services and psychological services
Cogollos‐de‐la‐Peña, R 2023 Spain	Uptake of physical rehabilitation services *To analyze social inequalities in the use and access of physiotherapy service and its clinical and socio-economic determinants in women diagnosed with breast cancer in the hospital network of Barcelona*	Cross-sectional cohort study (2235) Questionnaire; Cohort registry (DAMA) Education Multivariate Poisson regression model	No information provided	-	Women with a higher level of education had increased access to physiotherapy*
Ekels, A 2024 The Netherlands	Uptake of psychosocial services; physical rehabilitation services *To investigate health care utilization among patients with hematologic malignancies and its association with socioeconomic position (SEP) and compare health care utilization with a cancer-free population*	Retrospective cohort study (2319) Questionnaire; Cohort registry (PROFILES) Income; education Binary logistic regression	No information provided (about supportive care)		Patients with a high educational level reported statistically significant higher use of additional physical and psychosocial care during the first 2 years after diagnosis, compared to patients with a low educational level. Patients with a high-income level reported significantly higher utilization of psychosocial care from 2 years after diagnosis, compared to those with a low-income level*
Author Year Country	Relationship to Supportive care type Purpose of study	Study design (*n*) Data source SEP measure Statistical method	Multivariable adjustment		Finding (between SEP and access/uptake)
			Education*	Income*	
Strömberg, U 2024 Sweden	Access to physical rehabilitation services *To investigate the association between socioeconomic factors and different aspects of prostate cancer care, considering both individual- and neighborhood-level variables*	Population based cohort study (6505) National registry data (PCBaSe; NPCR) Education; income Multivariate cox regression models	Adjusted for: age, T stage, Gleason score, number of positive biopsy scores, prostate-specific antigen value, healthcare region at the time of diagnosis, individual/neighborhood-level socioeconomic variables		A statistically significant association for individual-level education (low versus high academic education) and rehabilitation was found*
Ziegler, E 2024 Germany	Access to psychosocial services *To examine which patient- and facility-specific factors are associated with patient-centered quality care delivery*	Cross-sectional study (1121) Questionnaire Education Binary Logistic regression	Adjusted for: age, sex, primary cancer type, years since diagnosis, decision making preference, health insurance, central contact person available	-	No significant associations between education and provision of psycho-oncological counseling were found

*Data for education or income shown for sex combined, unless indicated otherwise

**Statistical significance is indicated as defined by the original study

### Access/uptake of supportive care and association with SEP

**Fig. 2:**
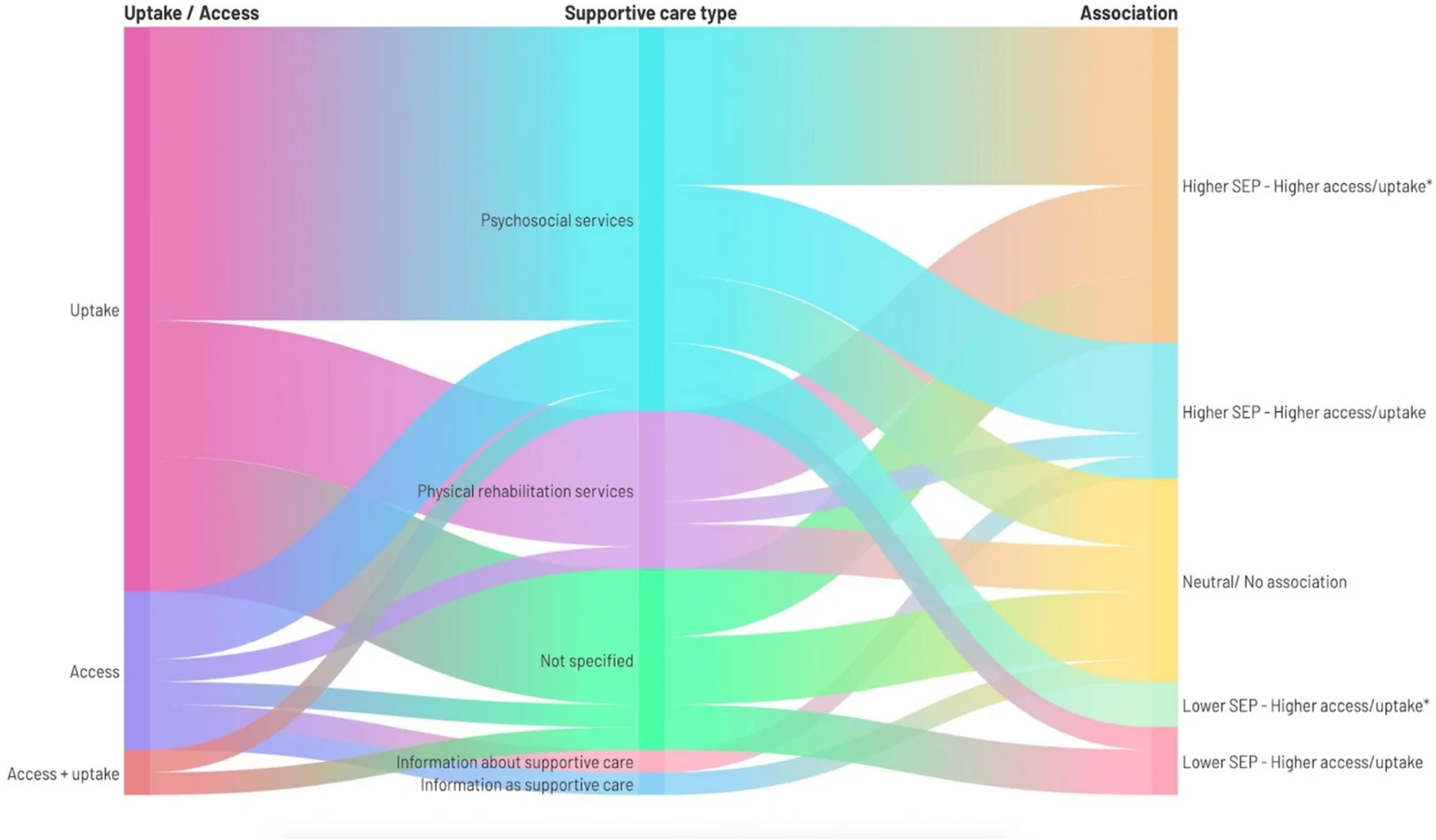
Sankey diagram illustrating the distribution of studies (*n* = 29) across access/uptake, type of supportive care, and observed association with SEP, with the width of each flow proportionate to the number of studies. Statistically significant associations are reported with an asterisk* in the right column

Figure 2 shows the evidence base formed by the included studies, depicting the proportions in either *access*, *uptake, or both* (left column), the type of care offered (middle column), and the observed association between supportive care use and SEP (right column). When reporting on observed associations, we differentiated between statistically significant and non-significant findings with an asterisk. Across all supportive care types, 12 studies found statistically significant associations between high SEP and increased access or uptake of supportive care, and 2 studies found statistically significant evidence for an association in the opposite direction.

 Fifteen studies *(with sample sizes ranging from 168 to 3704)* did not report statistically significant associations. Of these, five showed a tendency towards increased access among individuals with higher SEP [51, 56–58, 62]; seven reported neutral or no discernible patterns [46, 49, 55, 63–65, 85]; and three showed a tendency towards increased access among individuals with lower SEP [50, 66, 88].

### Psychosocial care

Seventeen studies involved psychosocial supportive care. *Access* to psychosocial care was assessed by 3 of these studies [52, 54, 58], whereas 14 studies assessed *uptake* in relation to SEP (Fig. 2). One of these studies reported on both access and uptake of psychosocial care [57]. Seven of the studies assessing psychosocial services found a significant association between high SEP and increased access to- or uptake of psychosocial care [47, 48, 52, 53, 59, 84, 87]. Five studies show a similar, but statistically non-significant tendency [51, 55–58]. Two studies found that lower SEP was linked to increased access, both focusing on social work or social support services [45, 54]. These were the only studies that examined social support services. As social support services are intended for especially vulnerable patients, these findings show their success in reaching the intended population [45, 55]. Three studies did not find any relevant association [46, 49, 55].

### Physical rehabilitation

Six out of seven studies reporting on physical rehabilitation services assessed uptake of supportive care [48, 49, 56, 59, 60, 87], and one assessed access to physical rehabilitation services [61]. Four of the studies found that high SEP was associated with increased uptake [48, 59–61]. Although not significant, the study by Korn et al. showed a similar tendency [56], and the study by Garmy et al. found a non-significant neutral association [49]. The study by Hofreuter-Gätgens showed non-significant associations in both directions, depending on the studied population (breast cancer or prostate cancer patients) [87].

### Other or unspecified supportive care types

Two Danish studies reported a significant association between high SEP and access to supportive care in the municipalities [43, 44], while the studies by Hensel et al., Garmy, Waldmann and Scherer-Trame et al. did not report any significant association between SEP and supportive care, nor did the data have any tendencies [49, 64, 65, 85]. Two studies reported on information receipt or access to information, yet they did not yield significant results [62, 63], although the study by Nicolaije et al. pointed towards a higher perceived receipt of information by those with a higher SEP [62].

## Discussion

### Summary

To the best of our knowledge, this review is the first to document access to- and uptake of supportive care for adult cancer patients in Europe, including both data on users and non-users. The majority of our studies (*n* = 29) show a positive association between high SEP and increased access or uptake of supportive care, either through statistically significant associations (*n* = 12) or tendencies of associations (*n* = 5). Five studies indicate an association between low SEP and increased access, with significant findings in the 2 studies assessing social support services or social work, indicating those more in need have higher access to social services [45, 54]. No studies reported on adherence to supportive care.

### Systemic factors

When comparing the associations found in the included studies, it should be taken into account that patient access and data thereof depends on how care is organized within health centers and institutions [46, 63]. As such, studies were able to interpret non-use of supportive care to varying extents. The study by Singer et al. for example, was conducted in a hospital certified by the German Cancer Society, which guarantees that patients are informed about available psychosocial services as standard practice [57]. As a result, this study included specific reasons for non-use of supportive care, whereas in the study by Zeissig et al., it was unclear whether non-utilization reflected services that were offered and declined or simply not offered at all [84]. In some cases, supportive care services are only recorded if they are delivered [84].

The studies conducted in Denmark show alternative pathways for access to supportive care provision: while specialized rehabilitation can be organized in the hospital setting, the responsibility for general rehabilitation lies in the municipalities [43, 44].

Although many European health models assume equal access through state financed insurance, several included studies noted that the option for supplementary or private insurance could still contribute to disparities in access to supportive care [55, 59, 60]. In instances where supplementary insurance is necessary for supportive care reimbursement, it could contribute to disparities in access to supportive care based on the insurance coverage individuals can afford. This review did not account for international differences in access to- and use of supportive care, where European health insurance models may range from fully subsidized care to varying degrees of coverage.

### Scope of chosen definitions and measures

Studies included in the review were concentrated in Northern and Western Europe, indicating that according to our definition of supportive care, this topic is understudied in Southern, Central and Eastern European regions. In general, access to supportive care may receive less attention in these countries, due to a focus on curative treatment or limited funding and availability [67]. It may also reflect differences in how supportive care is conceptualized and delivered across Europe. Alternatively, it could be explained by different approaches to supportive care provision in these regions (e.g. informally by family or support networks) [68, 69], which is a potential reflection of collectivist cultural values where familial responsibility has a central role [68, 70–74]. However, informal care does not constitute a formal supportive care service model, and was therefore beyond the scope of this review.

Our literature search was limited to objective measures of resources as a reflection of SEP. However, other relevant conceptualizations of SEP capture social, cultural, and subjective sensitivities that may influence if and how an individual receives care. In particular, Subjective Socioeconomic Status (SSS) assesses an individual’s experience and perception of where they stand relative to their environment, and has been shown to strongly predict health outcomes [21, 75–78]. SSS is associated with psychological factors such as a sense of control or future orientation, and, like with SEP, can compound with other patient characteristics such as levels of physical activity, health literacy, or social support, potentially affecting whether supportive care needs are met [47, 51, 53, 55, 63]. We suggest that future research initiatives consider including SSS when examining supportive care outcomes, which would require collecting patient reported data.

Additionally, measures of social stratification such as migration status or ethnicity can provide insight into structural barriers that affect supportive care access or uptake, yet these are not recorded consistently throughout Europe due to legal and ethical constraints [77, 81]. These barriers might affect access to care at a deeper level through reduced social capital or diverging cultural values [82, 83]. The studies by Singer et al. and Noeres et al. were the only studies to include a social stratification measure in assessing the association between migration status and uptake of psychosocial services [57, 86]. No significant results were found, but this was likely due to underrepresentation of individuals with a migration background in both studies.

### Methodology and reporting

Studies varied in methodology and the amount of information that was provided to describe their methods. The majority of studies described what the assessed supportive care consisted of [49, 51, 61] and included examples [48, 56, 59, 87], while other studies disclosed the setting in which the supportive care was provided [43, 44, 64, 65].

Continuous SEP measures (i.e. income and education) were often categorized into arbitrary categories, such as quartiles or sample specific categories. As this does not necessarily reflect absolute distributions, this limited comparability across studies.

The SEP measure was treated as a central determinant in a number of the studies, yet most studies used an exploratory approach to assess which variables, including SEP, are associated with supportive care usage. The mechanistic pathways were not always elaborated on in the analysis. For instance, five studies did not specify whether estimates were adjusted, and if so, for which variables they were adjusted, leaving ambiguity regarding whether observed effects were causal, influenced by potential confounding factors or mediation processes, or if they were simply associated with SEP. This lack of clarity limits the interpretability of findings, as unadjusted intergroup comparisons may overstate or understate the true relationship between SEP and supportive care access by failing to account for other influencing variables. Also, estimands may implicitly differ across studies (i.e. direct unmediated versus total effects).

The included cross-sectional studies listed the reliance on self-reporting or patient recall among the methodological limitations [60, 63], as well as lack of information on non-responders in questionnaire studies [50, 62]. Multiple studies reported generalizability issues, with an overrepresentation of women [48, 49], patients who are younger [46], patients with higher level of education or income [46–48, 58], patients with certain treatments [45], patients with a better prognosis [85], patients who have private health insurance [45], or patients in better physical condition [51].

### Limitations

This scoping review has several limitations. This review included literature up to May 2024, which entails that newer studies may have been published in the meantime. However, it is unlikely that engagement with supportive care in relation to SEP has dramatically changed over the course of one year, although methods may have improved since then. Studies that used composite measures to assess SEP were excluded if they contained variables that were not among our predefined SEP measures. Although this provided clarity, it may also have resulted in the exclusion of studies with legitimate assessments of other objective SEP markers. A methodological limitation was that while our search strategy focused on supportive care, it did not explicitly include search terms encompassing specific symptoms (i.e. fatigue) or highly specific symptom-relief care forms (i.e., lymph drainage). This entails that if eligible studies did not include the more general terms that were included in our search string, these would not have been identified. Although symptom relief studies, such as those assessing pain relief, often fall under the pharmaceutical realm and ultimately none of the pain-relief studies identified by our search met our inclusion criteria, future reviews should consider explicitly adding such search terms in their search criteria to increase sensitivity.

### Future research

We recommend future studies to assess the relationship between access to supportive care and SEP as their primary outcome, and to broaden the scope of interest to needs and willingness to receiving supportive care, as well as adherence rates following uptake in relation to the discussed SEP/SSS markers. Broadening the scope to include the full timeline of supportive care provision will contribute to understanding in which phase the provision of supportive care falls short. An understanding of how supportive care is organized across different European countries, and the premise under which their healthcare systems operate, would contribute to understanding different pathways of care. Furthermore, research initiatives reporting on supportive care and its socioeconomic correlates should include sufficient information on the type of supportive care being assessed, and in which setting it is offered. The ability to conduct thorough research on this topic, however, relies on the routine collection of data, including whether supportive care is offered, the types of supportive care provided, the pathway through which it is delivered, and patient characteristics (some of which may be sensitive in nature, such as migration status). Sensitive information should be collected using ethically sound methods, such as self-reporting after informed consent is collected, and questions should be carefully framed to ensure participants feel protected and properly understand the question. However, data collection on ethnicity or migration status may be prohibited by local legislation. Sufficient information on whether estimates are adjusted, and if so, why and for which factors, should be reported. We recommend that the relationship between individuals and access to care is studied in relation to subjective socioeconomic status (SSS) and measures of social stratification in the future.

## Conclusions and implications

This review provides the current state of knowledge on access to- and uptake of supportive care for cancer patients in Europe. Research on the association of SEP with access and uptake of supportive care is increasing but still limited. Methodology is widely varying and mostly exploratory. Still, a substantial proportion (*n* = 12) of the studies included in this review (*n* = 29) demonstrate a significant association between high SEP and increased access to- or uptake of supportive care across different care provision contexts in Europe. This points towards relative inaccessibility of supportive care and its potential to improve quality of life, to those in lower socioeconomic positions in the included countries. The provision of supportive care within in the oncology domain depends on infrastructural, contextual and patient-specific factors. The observed inequities call for an increased understanding of the pathways along which supportive care is offered across Europe, and how it might fail to reach socially and economically vulnerable patients who need it most. This should be a focus point for future research. Addressing disparities could involve developing health policies, integrating supportive care into clinical care guidelines, or outreach programs targeting lower SEP groups.

## Supplementary Information

Below is the link to the electronic supplementary material.

Supplementary file1 (DOCX 27 KB)

Supplementary file2 (DOCX 63 KB)

## Data Availability

No datasets were generated or analysed during the current study.
