# Supplementary material for: Socioeconomic position and access to supportive cancer care in Europe: a scoping review of literature from 2000 to 2024
Source: J Cancer Surviv. 2025 Oct 7;20(5):2649–67. doi: 10.1007/s11764-025-01904-3 (PMC13723617; doi:10.1007/s11764-025-01904-3)
Supplement: Supplementary file 1 [file 11764_2025_1904_MOESM1_ESM.docx]

**Appendix A**. The complete search strategy conducted through PubMed, CINAHL, EMBASE, and PsycINFO.

**Medline** via Ovid

| **#** | **Query** |
| --- | --- |
| 1 | exp neoplasms/ or "cancer survivors"/ |
| 2 | (cancer* or tumo?r? or neoplas* or malignan* or metasta* or adenocarcinom* or adenoma* or blastoma* or carcinom* or carcinosarcoma* or cholangiocarcinom* or chordoma* or germinoma* or gonadoblastoma* or hepatoblastoma* or "hodgkin?s disease" or "hodgkins disease" or leukemi* or leukaemi* or lymphangioma* or lymphangiomyoma* or lymphangiosarcoma* or lymphom* or melanom* or meningioma* or mesenchymoma* or mesonephroma* or myeloma or neuroma* or nsclc or paraneoplastic or plasmacytoma* or sarcoma* or teratocarcinoma* or teratoma* or oncogen* or oncolog*).ti,ab,kf. |
| 3 | or/1-2 [cancer] |
| 4 | exp europe/ |
| 5 | (europ* or andorra? or austria? or balkan? or belgium or belgian or benelux or albania? or baltic or estonia? or latvia? or lithuania? or bosnia? or herzegovinia? or bulgaria? or croatia? or czech* or hungar* or kosov* or moldova? or montenegro or montenegrin or poland or polish or pole or belarus* or macedonia* or romania? or russia? or serbia? or slovak* or slovenia? or slovene or ukrain* or france or french or german? or gibraltar* or greece or greek or hellene or ireland or eire or irish or italy or italian or liechtenstein* or luxembourg* or mediterranean or monaco or monegasque or monacan or netherlands or holland or dutch or portugal or portuguese or "san marino" or sammarinese or scandinavia? or denmark or danish or finland or finnish or finn or iceland* or norway or norwegian or sweden or swedish or spain or spanish or switzerland or swiss or transcaucasia or armenia? or azerbaijan? or azeri or georgia? or "united kingdom" or UK or britain or british or guernsey* or england or "northern ireland" or scotland or scots or scottish or wales or welsh or vatican*).ti,ab,kf. |
| 6 | or/4-5 [europe] |
| 7 | exp "Socioeconomic Factors"/ or "sociodemographic factors"/ or "healthcare disparities"/ |
| 8 | ("social inequalit*" or "social equalit*" or "social disparit*" or "social injustice" or literacy or illiteracy or income? or savings or pensions or "retirement benefit?" or salaries or salary or wage? or "pay equit*" or "medical indigenc*" or occupation? or vocation? or "career mobilit*" or "job ladder?" or povert* or indigen* or "low income" or "social change?" or "social impact?" or "social development?" or "social class*" or "social gradient?" or "middle class" or "lower class" or caste? or "social mobilit*" or "social condition?" or "living condition?" or "postal code?" or "area code?" or "zip code?" or slum? or ghetto?).ti,ab,kf. |
| 9 | (((socioeconomic* or "socio-economic*" or "socio-demographic*" or sociodemographic*) adj3 (factor? or characteristic? or status or position? or difference? or inequalit* or adversit* or resource? or condition? or parameter?)) or (standard adj3 living) or (economic adj3 (factor? or characteristic? or status or position or inequalit* or adversit* or resource? or condition?)) or (economic adj3 (stabilit* or instabilit* or insecurit*)) or ((education* or academic*) adj3 (level? or achievement* or status)) or ((healthcare or "health care") adj3 (disparit* or inequalit*))).ti,ab,kf. |
| 10 | (social adj1 (economic or demographic*) adj3 (factor? or characteristic? or status or position? or difference? or inequalit* or adversit* or resource? or condition? or parameter?)).ti,ab,kf. |
| 11 | or/7-10 [SES] |
| 12 | exp rehabilitation/ or exp "exercise therapy"/ or exp "physical therapy modalities"/ or exp "diet therapy"/ or exp aftercare/ or "Patient Education as Topic"/ or exp "social work"/ or "psychosocial support systems"/ or exp counseling/ or exp psychotherapy/ or "return to work"/ or "occupational therapy"/ or exp "Continuity of Patient Care"/ or exp "episode of care"/ or exp "long term care"/ |
| 13 | ("supportive care" or rehabilitat* or "re-habilitat*" or prerehabilitat* or "pre-rehabilitat*" or prehabilitat* or "pre-habilitat*" or "allied health care" or "physical therap*" or physiotherap* or "exercise therap*" or exercise or physiotherap* or "physical therap*" or kinesiotherap* or aftercare or "after care" or "after treatment?" or "follow-up care?" or "social work" or "psychosocial support" or "psychosocial care" or counseling or psychotherap* or psychologist? or "return to work" or "back to work" or "occupational therap*" or ergotherap* or "long term care").ti,ab,kf. |
| 14 | ((exercise? adj3 (therap* or remedial)) or ((nutrition* or diet*) adj3 (therap* or advice or intervention? or treatment?)) or (education* adj3 (care or intervention* or therap* or patient? or material)) or ("nurse led" adj3 (care or intervention* or therap*)) or (social adj3 intervention*) or ("patient care" adj3 continuity) or (care adj3 continuum) or (patient? adj3 discharg*) or (patient? adj3 (handoff? or "hand over")) or ((patient? or care) adj3 (transfer? or transition?)) or (care adj3 episode?)).ti,ab,kf. |
| 15 | or/12-14 [therapy] |
| 16 | 3 and 6 and 11 and 15 [cancer AND europe AND SES AND therapy] |

**Embase** via embase.com

| **#** | **Query** |
| --- | --- |
| 1 | 'neoplasm'/exp OR 'cancer survivor'/exp |
| 2 | (cancer* OR tumo$r$ OR neoplas* OR malignan* OR metasta* OR adenocarcinom* OR adenoma* OR blastoma* OR carcinom* OR carcinosarcoma* OR cholangiocarcinom* OR chordoma* OR germinoma* OR gonadoblastoma* OR hepatoblastoma* OR 'hodgkin$s disease' or 'hodgkins disease' OR leukemi* OR leukaemi* OR lymphangioma* OR lymphangiomyoma* OR lymphangiosarcoma* OR lymphom* OR melanom* OR meningioma* OR mesenchymoma* OR mesonephroma* OR myeloma OR neuroma* OR nsclc OR paraneoplastic OR plasmacytoma* OR sarcoma* OR teratocarcinoma* OR teratoma* OR oncogen* OR oncolog*):ti,ab,kw |
| 3 | #1 OR #2 |
| 4 | 'europe'/exp |
| 5 | (europ* OR andorra$ OR austria$ OR balkan$ OR belgium OR belgian OR benelux OR albania$ OR baltic OR estonia$ OR latvia$ OR lithuania$ OR bosnia$ OR herzegovinia$ OR bulgaria$ OR croatia$ OR czech* OR hungar* OR kosov* OR moldova$ OR montenegro OR montenegrin OR poland OR polish OR pole OR belarus* OR macedonia* OR romania$ OR russia$ OR serbia$ OR slovak* OR slovenia$ OR slovene OR ukrain* OR france OR french OR german$ OR gibraltar* OR greece OR greek OR hellene OR ireland OR eire OR irish OR italy OR italian OR liechtenstein* OR luxembourg* OR mediterranean OR monaco OR monegasque OR monacan OR netherlands OR holland OR dutch OR portugal OR portuguese OR 'san marino' OR sammarinese OR scandinavia$ OR denmark OR danish OR finland OR finnish OR finn OR iceland* OR norway OR norwegian OR sweden OR swedish OR spain OR spanish OR switzerland OR swiss OR transcaucasia OR armenia$ OR azerbaijan$ OR azeri OR georgia$ OR 'united kingdom' OR UK OR britain OR british OR guernsey* OR england OR 'northern ireland' OR scotland OR scots OR scottish OR wales OR welsh OR vatican*):ti,ab,kw |
| 6 | #4 OR #5 |
| 7 | 'socioeconomics'/de OR 'economic status'/de OR 'household economic status'/de OR 'educational status'/exp OR 'externality'/exp OR 'housing instability'/de OR 'income group'/exp OR 'pay equity'/de OR 'poverty'/exp OR 'socioeconomic vulnerability'/exp OR 'health care disparity'/exp |
| 8 | ('social inequalit*' OR 'social equalit*' OR 'social disparit*' OR 'social injustice' OR literacy OR illiteracy OR income$ OR savings OR pensions OR 'retirement benefit$' OR salaries OR salary OR wage$ OR 'pay equit*' OR 'medical indigenc*' OR occupation$ OR vocation$ OR 'career mobilit*' OR 'job ladder?' OR povert* OR indigen* OR 'low income' OR 'social change$' OR 'social impact$' OR 'social development$' OR 'social class*' OR 'social gradient$' OR 'middle class' OR 'lower class' OR caste$ OR 'social mobilit*' OR 'social condition$' OR 'living condition$' OR 'postal code$' OR 'area code$' OR 'zip code$' OR slum$ OR ghetto$):ti,ab,kw |
| 9 | (((socioeconomic* OR 'socio-economic*' OR 'socio-demographic*' OR sociodemographic*) NEAR/3 (factor$ OR characteristic$ OR status OR position$ OR difference$ OR inequalit* OR adversit* OR resource$ OR condition$ OR parameter$)):ti,ab,kw) OR ((standard NEAR/3 living):ti,ab,kw) OR ((economic NEAR/3 (factor$ OR characteristic$ OR status OR position OR inequalit* OR adversit* OR resource$ OR condition$ OR stabilit* OR instabilit* OR insecurit*)):ti,ab,kw) OR (((education* OR academic*) NEAR/3 (level$ OR achievement* OR status)):ti,ab,kw) OR (((healthcare OR 'health care') NEAR/3 (disparit* OR inequalit*)):ti,ab,kw) |
| 10 | (social NEAR/1 (economic OR demographic*) NEAR/3 (factor$ OR characteristic$ OR status OR position$ OR difference$ OR inequalit* OR adversit* OR resource$ OR condition$ OR parameter$)):ti,ab,kw |
| 11 | #7 OR #8 OR #9 OR #10 |
| 12 | 'supportive care'/de OR 'rehabilitation'/exp OR 'kinesiotherapy'/exp OR 'physiotherapy'/exp OR 'diet therapy'/exp OR 'aftercare'/de OR 'patient education'/exp OR 'nurse led intervention'/de OR 'social work'/exp OR 'psychosocial care'/exp OR 'counseling'/exp OR 'return to work'/exp OR 'occupational therapy'/exp OR 'hospital discharge'/exp OR 'long term care'/de |
| 13 | 'supportive care':ti,ab,kw OR rehabilitat*:ti,ab,kw OR 're-habilitat*':ti,ab,kw OR prerehabilitat*:ti,ab,kw OR 'pre-rehabilitat*':ti,ab,kw OR prehabilitat*:ti,ab,kw OR 'pre-habilitat*':ti,ab,kw OR 'allied health care':ti,ab,kw OR exercise:ti,ab,kw OR physiotherap*:ti,ab,kw OR 'physical therap*':ti,ab,kw OR kinesiotherap*:ti,ab,kw OR 'after treatment?':ti,ab,kw OR 'follow-up care?':ti,ab,kw OR 'social work':ti,ab,kw OR 'psychosocial support':ti,ab,kw OR 'psychosocial care':ti,ab,kw OR counseling:ti,ab,kw OR psychotherap*:ti,ab,kw OR psychologist$:ti,ab,kw OR 'return to work':ti,ab,kw OR 'back to work':ti,ab,kw OR 'occupational therap*':ti,ab,kw OR ergotherap*:ti,ab,kw OR aftercare:ti,ab,kw OR 'after care':ti,ab,kw OR 'follow up care':ti,ab,kw OR 'long term care':ti,ab,kw |
| 14 | ((exercise$ NEAR/3 (therap* OR remedial)):ti,ab,kw) OR (((nutrition* OR diet*) NEAR/3 (therap* OR advice OR intervention$ OR treatment?)):ti,ab,kw) OR ((education* NEAR/3 (care OR intervention* OR therap* OR patient$ OR material)):ti,ab,kw) OR (('nurse led' NEAR/3 (care OR intervention* OR therap*)):ti,ab,kw) OR ((social NEAR/3 intervention*):ti,ab,kw) OR (('patient care' NEAR/3 continuity):ti,ab,kw) OR ((care NEAR/3 continuum):ti,ab,kw) OR ((patient$ NEAR/3 discharg*):ti,ab,kw) OR ((patient$ NEAR/3 (handoff$ OR 'hand over')):ti,ab,kw) OR (((patient$ OR care) NEAR/3 (transfer$ OR transition$)):ti,ab,kw) OR ((care NEAR/3 episode$):ti,ab,kw) |
| 15 | #12 OR #13 OR #14 |
| 16 | #3 AND #6 AND #11 AND #15 |
| 17 | #16 NOT ('conference abstract'/it OR 'conference review'/it) |

**Scopus**

**Query**

(

TITLE-ABS(cancer* OR tumor* OR tumour* OR neoplas* OR malignan* OR metasta* OR adenocarcinom* OR adenoma* OR blastoma* OR carcinom* OR carcinosarcoma* OR cholangiocarcinom* OR chordoma* OR germinoma* OR gonadoblastoma* OR hepatoblastoma* OR "hodgkin's disease" or "hodgkins disease" OR leukemi* OR leukaemi* OR lymphangioma* OR lymphangiomyoma* OR lymphangiosarcoma* OR lymphom* OR melanom* OR meningioma* OR mesenchymoma* OR mesonephroma* OR myeloma OR neuroma* OR nsclc OR paraneoplastic OR plasmacytoma* OR sarcoma* OR teratocarcinoma* OR teratoma* OR oncogen* OR oncolog*)

OR AUTHKEY(cancer* OR tumor* OR tumour* OR neoplas* OR malignan* OR metasta* OR adenocarcinom* OR adenoma* OR blastoma* OR carcinom* OR carcinosarcoma* OR cholangiocarcinom* OR chordoma* OR germinoma* OR gonadoblastoma* OR hepatoblastoma* OR "hodgkin's disease" or "hodgkins disease" OR leukemi* OR leukaemi* OR lymphangioma* OR lymphangiomyoma* OR lymphangiosarcoma* OR lymphom* OR melanom* OR meningioma* OR mesenchymoma* OR mesonephroma* OR myeloma OR neuroma* OR nsclc OR paraneoplastic OR plasmacytoma* OR sarcoma* OR teratocarcinoma* OR teratoma* OR oncogen* OR oncolog*)

)

AND (

TITLE-ABS(europ* OR andorra* OR austria* OR balkan* OR belgium OR belgian OR benelux OR albania* OR baltic OR estonia* OR latvia* OR lithuania* OR bosnia* OR herzegovinia* OR bulgaria* OR croatia* OR czech* OR hungar* OR kosov* OR moldova* OR montenegro OR montenegrin OR poland OR polish OR pole OR belarus* OR macedonia* OR romania* OR russia* OR serbia* OR slovak* OR slovenia* OR slovene OR ukrain* OR france OR french OR german* OR gibraltar* OR greece OR greek OR hellene OR ireland OR eire OR irish OR italy OR italian OR liechtenstein* OR luxembourg* OR mediterranean OR monaco OR monegasque OR monacan OR netherlands OR holland OR dutch OR portugal OR portuguese OR "san marino" OR sammarinese OR scandinavia* OR denmark OR danish OR finland OR finnish OR finn OR iceland* OR norway OR norwegian OR sweden OR swedish OR spain OR spanish OR switzerland OR swiss OR transcaucasia OR armenia* OR azerbaijan* OR azeri OR georgia* OR "united kingdom" OR UK OR british OR britain OR guernsey* OR england OR "northern ireland" OR scotland OR scots OR scottish OR wales OR welsh OR vatican*)

OR AUTHKEY(europ* OR andorra* OR austria* OR balkan* OR belgium OR belgian OR benelux OR albania* OR baltic OR estonia* OR latvia* OR lithuania* OR bosnia* OR herzegovinia* OR bulgaria* OR croatia* OR czech* OR hungar* OR kosov* OR moldova* OR montenegro OR montenegrin OR poland OR polish OR pole OR belarus* OR macedonia* OR romania* OR russia* OR serbia* OR slovak* OR slovenia* OR slovene OR ukrain* OR france OR french OR german* OR gibraltar* OR greece OR greek OR hellene OR ireland OR eire OR irish OR italy OR italian OR liechtenstein* OR luxembourg* OR mediterranean OR monaco OR monegasque OR monacan OR netherlands OR holland OR dutch OR portugal OR portuguese OR "san marino" OR sammarinese OR scandinavia* OR denmark OR danish OR finland OR finnish OR finn OR iceland* OR norway OR norwegian OR sweden OR swedish OR spain OR spanish OR switzerland OR swiss OR transcaucasia OR armenia* OR azerbaijan* OR azeri OR georgia* OR "united kingdom" OR UK OR british OR britain OR guernsey* OR england OR "northern ireland" OR scotland OR scots OR scottish OR wales OR welsh OR vatican*)

)

AND (

TITLE-ABS(("social inequalit*" OR "social equalit*" OR "social disparit*" OR "social injustice" OR literacy OR illiteracy OR income* OR savings OR pensions OR "retirement benefit*" OR salaries OR salary OR wage* OR "pay equit*" OR "medical indigenc*" OR occupation* OR vocation* OR "career mobilit*" OR "job ladder*" OR povert* OR indigen* OR "low income" OR "social change*" OR "social impact*" OR "social development*" OR "social class*" OR "social gradient*" OR "middle class" OR "lower class" OR caste* OR "social mobilit*" OR "social condition*" OR "living condition*" OR "postal code*" OR "area code*" OR "zip code*" OR slum* OR ghetto*) OR (((socioeconomic* OR "socio-economic*" OR "socio-demographic*" OR sociodemographic*) W/2 (factor* OR characteristic* OR status OR position* OR difference* OR inequalit* OR adversit* OR resource* OR condition* OR parameter*)) OR (standard W/2 living) OR (economic W/2 (factor* OR characteristic* OR status OR position OR inequalit* OR adversit* OR resource* OR condition*)) OR (economic W/2 (stabilit* OR instabilit* OR insecurit*)) OR ((education* OR academic*) W/2 (level* OR achievement* OR status)) OR ((healthcare OR "health care") W/2 (disparit* OR inequalit*))) OR (social W/1 (economic OR demographic*) W/2 (factor* OR characteristic* OR status OR position* OR difference* OR inequalit* OR adversit* OR resource* OR condition* OR parameter*)))

OR AUTHKEY(("social inequalit*" OR "social equalit*" OR "social disparit*" OR "social injustice" OR literacy OR illiteracy OR income* OR savings OR pensions OR "retirement benefit*" OR salaries OR salary OR wage* OR "pay equit*" OR "medical indigenc*" OR occupation* OR vocation* OR "career mobilit*" OR "job ladder*" OR povert* OR indigen* OR "low income" OR "social change*" OR "social impact*" OR "social development*" OR "social class*" OR "social gradient*" OR "middle class" OR "lower class" OR caste* OR "social mobilit*" OR "social condition*" OR "living condition*" OR "postal code*" OR "area code*" OR "zip code*" OR slum* OR ghetto*) OR (((socioeconomic* OR "socio-economic*" OR "socio-demographic*" OR sociodemographic*) W/2 (factor* OR characteristic* OR status OR position* OR difference* OR inequalit* OR adversit* OR resource* OR condition* OR parameter*)) OR (standard W/2 living) OR (economic W/2 (factor* OR characteristic* OR status OR position OR inequalit* OR adversit* OR resource* OR condition*)) OR (economic W/2 (stabilit* OR instabilit* OR insecurit*)) OR ((education* OR academic*) W/2 (level* OR achievement* OR status)) OR ((healthcare OR "health care") W/2 (disparit* OR inequalit*))) OR (social W/1 (economic OR demographic*) W/2 (factor* OR characteristic* OR status OR position* OR difference* OR inequalit* OR adversit* OR resource* OR condition* OR parameter*)))

)

AND (

TITLE-ABS(("supportive care" OR rehabilitat* OR "re-habilitat*" OR prerehabilitat* OR "pre-rehabilitat*" OR prehabilitat* OR "pre-habilitat*" OR "allied health care" OR "physical therap*" OR physiotherap* OR "exercise therap*" OR exercise OR physiotherap* OR "physical therap*" OR kinesiotherap* OR aftercare OR "after care" OR "after treatment*" OR "follow-up care*" OR "social work" OR "psychosocial support" OR "psychosocial care" OR counseling OR psychotherap* OR psychologist* OR "return to work" OR "back to work" OR "occupational therap*" OR ergotherap* OR "long term care") OR ((exercise* W/2 (therap* OR remedial)) OR ((nutrition* OR diet*) W/2 (therap* OR advice OR intervention* OR treatment*)) OR (education* W/2 (care OR intervention* OR therap* OR patient* OR material)) OR ("nurse led" W/2 (care OR intervention* OR therap*)) OR (social W/2 intervention*) OR ("patient care" W/2 continuity) OR (care W/2 continuum) OR (patient* W/2 discharg*) OR (patient* W/2 (handoff* OR "hand over")) OR ((patient* OR care) W/2 (transfer* OR transition*)) OR (care W/2 episode*)))

OR AUTHKEY(("supportive care" OR rehabilitat* OR prerehabilitat* OR "allied health care" OR "physical therap*" OR physiotherap* OR "exercise therap*" OR exercise OR physiotherap* OR "physical therap*" OR kinesiotherap* OR aftercare OR "after care" OR "after treatment*" OR "follow-up care*" OR "social work" OR "psychosocial support" OR "psychosocial care" OR counseling OR psychotherap* OR psychologist* OR "return to work" OR "back to work" OR "occupational therap*" OR ergotherap* OR "long term care") OR ((exercise* W/2 (therap* OR remedial)) OR ((nutrition* OR diet*) W/2 (therap* OR advice OR intervention* OR treatment*)) OR (education* W/2 (care OR intervention* OR therap* OR patient* OR material)) OR ("nurse led" W/2 (care OR intervention* OR therap*)) OR (social W/2 intervention*) OR ("patient care" W/2 continuity) OR (care W/2 continuum) OR (patient* W/2 discharg*) OR (patient* W/2 (handoff* OR "hand over")) OR ((patient* OR care) W/2 (transfer* OR transition*)) OR (care W/2 episode*)))

)

**PsycInfo** via Ovid

| **#** | **Query** |
| --- | --- |
| 1 | neoplasms.sh. |
| 2 | (cancer* or tumo?r? or neoplas* or malignan* or metasta* or adenocarcinom* or adenoma* or blastoma* or carcinom* or carcinosarcoma* or cholangiocarcinom* or chordoma* or germinoma* or gonadoblastoma* or hepatoblastoma* or "hodgkin?s disease" or "hodgkins disease" or leukemi* or leukaemi* or lymphangioma* or lymphangiomyoma* or lymphangiosarcoma* or lymphom* or melanom* or meningioma* or mesenchymoma* or mesonephroma* or myeloma or neuroma* or nsclc or paraneoplastic or plasmacytoma* or sarcoma* or teratocarcinoma* or teratoma* or oncogen* or oncolog*).ti,ab,id. |
| 3 | OR/1-2 [cancer] |
| 4 | (europ* or andorra? or austria? or balkan? or belgium or belgian or benelux or albania? or baltic or estonia? or latvia? or lithuania? or bosnia? or herzegovinia? or bulgaria? or croatia? or czech* or hungar* or kosov* or moldova? or montenegro or montenegrin or poland or polish or pole or belarus* or macedonia* or romania? or russia? or serbia? or slovak* or slovenia? or slovene or ukrain* or france or french or german? or gibraltar* or greece or greek or hellene or ireland or eire or irish or italy or italian or liechtenstein* or luxembourg* or mediterranean or monaco or monegasque or monacan or netherlands or holland or dutch or portugal or portuguese or "san marino" or sammarinese or scandinavia? or denmark or danish or finland or finnish or finn or iceland* or norway or norwegian or sweden or swedish or spain or spanish or switzerland or swiss or transcaucasia or armenia? or azerbaijan? or azeri or georgia? or "united kingdom" or UK or britain or british or guernsey* or england or "northern ireland" or scotland or scots or scottish or wales or welsh or vatican*).ti,ab,id. [Europe] |
| 5 | "socioeconomic status".sh. |
| 6 | ("social inequalit*" or "social equalit*" or "social disparit*" or "social injustice" or literacy or illiteracy or income? or savings or pensions or "retirement benefit?" or salaries or salary or wage? or "pay equit*" or "medical indigenc*" or occupation? or vocation? or "career mobilit*" or "job ladder?" or povert* or indigen* or "low income" or "social change?" or "social impact?" or "social development?" or "social class*" or "social gradient?" or "middle class" or "lower class" or caste? or "social mobilit*" or "social condition?" or "living condition?" or "postal code?" or "area code?" or "zip code?" or slum? or ghetto?).ti,ab,id. |
| 7 | (((socioeconomic* OR "socio-economic*" OR "socio-demographic*" OR sociodemographic*) ADJ3 (factor? OR characteristic? OR status OR position? OR difference? OR inequalit* OR adversit* OR resource? OR condition* OR parameter?)) OR (standard ADJ3 living) OR (economic ADJ3 (factor? OR characteristic? OR status OR position OR inequalit* OR adversit* OR resource? OR condition?)) OR (economic ADJ3 (stabilit* OR instabilit* OR insecurit*)) OR ((education* OR academic*) ADJ3 (level? OR achievement* OR status)) OR ((healthcare OR "health care") ADJ3 (disparit* OR inequalit*))).ti,ab,id. |
| 8 | (social ADJ1 (economic OR demographic*) ADJ3 (factor? or characteristic? or status or position? or difference? or inequalit* or adversit* or resource? or condition? or parameter?)).ti,ab,id. |
| 9 | OR/5-8 [SES] |
| 10 | (rehabilitation OR "physical therapy" OR "occupational therapy" OR "dietary treatment" OR aftercare OR "client education" OR "Psychosocial Interventions" OR counseling OR "rehabilitation counseling" OR psychotherapy OR "long term care").sh. |
| 11 | ("supportive care" or rehabilitat* or "re-habilitat*" or prerehabilitat* or "pre-rehabilitat*" or prehabilitat* or "pre-habilitat*" or "allied health care" or "physical therap*" or physiotherap* or "exercise therap*" or exercise or physiotherap* or "physical therap*" or kinesiotherap* or aftercare or "after care" or "after treatment?" or "follow-up care?" or "social work" or "psychosocial support" or "psychosocial care" or counseling or psychotherap* or psychologist? or "return to work" or "back to work" or "occupational therap*" or ergotherap* or "long term care").ti,ab,id. |
| 12 | ((exercise? ADJ3 (therap* OR remedial)) OR ((nutrition* OR diet*) ADJ3 (therap* OR advice OR intervention?)) OR (education* ADJ3 (care OR intervention* OR therap* OR patient? OR material)) OR ("nurse led" ADJ3 (care OR intervention* OR therap*)) OR (social ADJ3 intervention*) OR ("patient care" ADJ3 continuity) OR (care ADJ3 continuum) OR (patient? ADJ3 discharg*) OR (patient? ADJ3 (handoff? OR "hand over")) OR ((patient? OR care) ADJ3 (transfer? OR transition?)) OR (care ADJ3 episode?)).ti,ab,id. |
| 13 | OR/10-12 [therapy] |
| 14 | 3 AND 4 AND 9 AND 13 [cancer AND Europe AND SES AND therapy] |

**CINAHL** via Ebsco

**Query**

( MH(neoplasms+) OR TI(cancer* OR tumo#r# OR neoplas* OR malignan* OR metasta* OR adenocarcinom* OR adenoma* OR blastoma* OR carcinom* OR carcinosarcoma* OR cholangiocarcinom* OR chordoma* OR germinoma* OR gonadoblastoma* OR hepatoblastoma* OR "hodgkin#s disease" or "hodgkins disease" OR leukemi* OR leukaemi* OR lymphangioma* OR lymphangiomyoma* OR lymphangiosarcoma* OR lymphom* OR melanom* OR meningioma* OR mesenchymoma* OR mesonephroma* OR myeloma OR neuroma* OR nsclc OR paraneoplastic OR plasmacytoma* OR sarcoma* OR teratocarcinoma* OR teratoma* OR oncogen* OR oncolog*) OR AB(cancer* OR tumo#r# OR neoplas* OR malignan* OR metasta* OR adenocarcinom* OR adenoma* OR blastoma* OR carcinom* OR carcinosarcoma* OR cholangiocarcinom* OR chordoma* OR germinoma* OR gonadoblastoma* OR hepatoblastoma* OR "hodgkin#s disease" or "hodgkins disease" OR leukemi* OR leukaemi* OR lymphangioma* OR lymphangiomyoma* OR lymphangiosarcoma* OR lymphom* OR melanom* OR meningioma* OR mesenchymoma* OR mesonephroma* OR myeloma OR neuroma* OR nsclc OR paraneoplastic OR plasmacytoma* OR sarcoma* OR teratocarcinoma* OR teratoma* OR oncogen* OR oncolog*) OR DE(cancer* OR tumo#r# OR neoplas* OR malignan* OR metasta* OR adenocarcinom* OR adenoma* OR blastoma* OR carcinom* OR carcinosarcoma* OR cholangiocarcinom* OR chordoma* OR germinoma* OR gonadoblastoma* OR hepatoblastoma* OR "hodgkin#s disease" or "hodgkins disease" OR leukemi* OR leukaemi* OR lymphangioma* OR lymphangiomyoma* OR lymphangiosarcoma* OR lymphom* OR melanom* OR meningioma* OR mesenchymoma* OR mesonephroma* OR myeloma OR neuroma* OR nsclc OR paraneoplastic OR plasmacytoma* OR sarcoma* OR teratocarcinoma* OR teratoma* OR oncogen* OR oncolog*) ) AND ( MH(europe+) OR TI(europ* OR andorra# OR austria# OR balkan# OR belgium OR belgian OR benelux OR albania# OR baltic OR estonia# OR latvia# OR lithuania# OR bosnia# OR herzegovinia# OR bulgaria# OR croatia# OR czech* OR hungar* OR kosov* OR moldova# OR montenegro OR montenegrin OR poland OR polish OR pole OR belarus* OR macedonia* OR romania# OR russia# OR serbia# OR slovak* OR slovenia# OR slovene OR ukrain* OR france OR french OR german# OR gibraltar* OR greece OR greek OR hellene OR ireland OR eire OR irish OR italy OR italian OR liechtenstein* OR luxembourg* OR mediterranean OR monaco OR monegasque OR monacan OR netherlands OR holland OR dutch OR portugal OR portuguese OR "san marino" OR sammarinese OR scandinavia# OR denmark OR danish OR finland OR finnish OR finn OR iceland* OR norway OR norwegian OR sweden OR swedish OR spain OR spanish OR switzerland OR swiss OR transcaucasia OR armenia# OR azerbaijan# OR azeri OR georgia# OR "united kingdom" OR UK OR british OR britain OR guernsey* OR england OR "northern ireland" OR scotland OR scots OR scottish OR wales OR welsh OR vatican*) OR AB(europ* OR andorra# OR austria# OR balkan# OR belgium OR belgian OR benelux OR albania# OR baltic OR estonia# OR latvia# OR lithuania# OR bosnia# OR herzegovinia# OR bulgaria# OR croatia# OR czech* OR hungar* OR kosov* OR moldova# OR montenegro OR montenegrin OR poland OR polish OR pole OR belarus* OR macedonia* OR romania# OR russia# OR serbia# OR slovak* OR slovenia# OR slovene OR ukrain* OR france OR french OR german# OR gibraltar* OR greece OR greek OR hellene OR ireland OR eire OR irish OR italy OR italian OR liechtenstein* OR luxembourg* OR mediterranean OR monaco OR monegasque OR monacan OR netherlands OR holland OR dutch OR portugal OR portuguese OR "san marino" OR sammarinese OR scandinavia# OR denmark OR danish OR finland OR finnish OR finn OR iceland* OR norway OR norwegian OR sweden OR swedish OR spain OR spanish OR switzerland OR swiss OR transcaucasia OR armenia# OR azerbaijan# OR azeri OR georgia# OR "united kingdom" OR UK OR british OR britain OR guernsey* OR england OR "northern ireland" OR scotland OR scots OR scottish OR wales OR welsh OR vatican*) OR DE(europ* OR andorra# OR austria# OR balkan# OR belgium OR belgian OR benelux OR albania# OR baltic OR estonia# OR latvia# OR lithuania# OR bosnia# OR herzegovinia# OR bulgaria# OR croatia# OR czech* OR hungar* OR kosov* OR moldova# OR montenegro OR montenegrin OR poland OR polish OR pole OR belarus* OR macedonia* OR romania# OR russia# OR serbia# OR slovak* OR slovenia# OR slovene OR ukrain* OR france OR french OR german# OR gibraltar* OR greece OR greek OR hellene OR ireland OR eire OR irish OR italy OR italian OR liechtenstein* OR luxembourg* OR mediterranean OR monaco OR monegasque OR monacan OR netherlands OR holland OR dutch OR portugal OR portuguese OR "san marino" OR sammarinese OR scandinavia# OR denmark OR danish OR finland OR finnish OR finn OR iceland* OR norway OR norwegian OR sweden OR swedish OR spain OR spanish OR switzerland OR swiss OR transcaucasia OR armenia# OR azerbaijan# OR azeri OR georgia# OR "united kingdom" OR UK OR british OR britain OR guernsey* OR england OR "northern ireland" OR scotland OR scots OR scottish OR wales OR welsh OR vatican*) ) AND ( MH("Socioeconomic Factors+" OR "Sociodemographic Factors" OR "Healthcare Disparities") OR TI(("social inequalit*" OR "social equalit*" OR "social disparit*" OR literacy OR illiteracy OR income# OR savings OR pensions OR "retirement benefit#" OR salaries OR salary OR wage# OR "pay equit*" OR "medical indigenc*" OR occupation# OR vocation# OR "career mobilit*" OR "job ladder#" OR povert* OR indigen* OR "low income" OR "social change#" OR "social impact#" OR "social development#" OR "social class*" OR "social gradient#" OR "middle class" OR "lower class" OR caste# OR "social mobilit*" OR "social condition#" OR "living condition#" OR "postal code#" OR "area code#" OR "zip code#" OR slum# OR ghetto#) OR (((socioeconomic* OR "socio-economic*" OR "socio-demographic*" OR sociodemographic*) N2 (factor# OR characteristic# OR status OR position# OR difference# OR inequalit* OR adversit* OR resource# OR condition# OR parameter#)) OR (standard N2 living) OR (economic N2 (factor# OR characteristic# OR status OR position OR inequalit* OR adversit* OR resource# OR condition#)) OR (economic N2 (stabilit* OR instabilit* OR insecurit*)) OR ((education* OR academic*) N2 (level# OR achievement* OR status)) OR ((healthcare OR "health care") N2 (disparit* OR inequalit*))) OR (social N2 (economic OR demographic*) N2 (factor# OR characteristic# OR status OR position# OR difference# OR inequalit* OR adversit* OR resource# OR condition# OR parameter#))) OR AB(("social inequalit*" OR "social equalit*" OR "social disparit*" OR literacy OR illiteracy OR income# OR savings OR pensions OR "retirement benefit#" OR salaries OR salary OR wage# OR "pay equit*" OR "medical indigenc*" OR occupation# OR vocation# OR "career mobilit*" OR "job ladder#" OR povert* OR indigen* OR "low income" OR "social change#" OR "social impact#" OR "social development#" OR "social class*" OR "social gradient#" OR "middle class" OR "lower class" OR caste# OR "social mobilit*" OR "social condition#" OR "living condition#" OR "postal code#" OR "area code#" OR "zip code#" OR slum# OR ghetto#) OR (((socioeconomic* OR "socio-economic*" OR "socio-demographic*" OR sociodemographic*) N2 (factor# OR characteristic# OR status OR position# OR difference# OR inequalit* OR adversit* OR resource# OR condition# OR parameter#)) OR (standard N2 living) OR (economic N2 (factor# OR characteristic# OR status OR position OR inequalit* OR adversit* OR resource# OR condition#)) OR (economic N2 (stabilit* OR instabilit* OR insecurit*)) OR ((education* OR academic*) N2 (level# OR achievement* OR status)) OR ((healthcare OR "health care") N2 (disparit* OR inequalit*))) OR (social N2 (economic OR demographic*) N2 (factor# OR characteristic# OR status OR position# OR difference# OR inequalit* OR adversit* OR resource# OR condition# OR parameter#))) OR DE(("social inequalit*" OR "social equalit*" OR "social disparit*" OR literacy OR illiteracy OR income# OR savings OR pensions OR "retirement benefit#" OR salaries OR salary OR wage# OR "pay equit*" OR "medical indigenc*" OR occupation# OR vocation# OR "career mobilit*" OR "job ladder#" OR povert* OR indigen* OR "low income" OR "social change#" OR "social impact#" OR "social development#" OR "social class*" OR "social gradient#" OR "middle class" OR "lower class" OR caste# OR "social mobilit*" OR "social condition#" OR "living condition#" OR "postal code#" OR "area code#" OR "zip code#" OR slum# OR ghetto#) OR (((socioeconomic* OR "socio-economic*" OR "socio-demographic*" OR sociodemographic*) N2 (factor# OR characteristic# OR status OR position# OR difference# OR inequalit* OR adversit* OR resource# OR condition# OR parameter#)) OR (standard N2 living) OR (economic N2 (factor# OR characteristic# OR status OR position OR inequalit* OR adversit* OR resource# OR condition#)) OR (economic N2 (stabilit* OR instabilit* OR insecurit*)) OR ((education* OR academic*) N2 (level# OR achievement* OR status)) OR ((healthcare OR "health care") N2 (disparit* OR inequalit*))) OR (social N2 (economic OR demographic*) N2 (factor# OR characteristic# OR status OR position# OR difference# OR inequalit* OR adversit* OR resource# OR condition# OR parameter#))) ) AND ( MH(Rehabilitation+ OR "diet therapy+" OR "after care" OR "Patient Education+" OR "social work+" OR "Support, Psychosocial+" OR "counseling+" OR "psychotherapy+" OR "occupational therapy" OR "continuity of patient care+" OR "long term care") OR TI(("supportive care" OR rehabilitat* OR "re-habilitat*" OR prerehabilitat* OR "pre-rehabilitat*" OR prehabilitat* OR "pre-habilitat*" OR "allied health care" OR "physical therap*" OR physiotherap* OR "exercise therap*" OR exercise OR physiotherap* OR "physical therap*" OR kinesiotherap* OR aftercare OR "after care" OR "after treatment#" OR "follow-up care#" OR "social work" OR "psychosocial support" OR "psychosocial care" OR counseling OR psychotherap* OR psychologist# OR "return to work" OR "back to work" OR "occupational therap*" OR ergotherap* OR "long term care") OR ((exercise# N2 (therap* OR remedial)) OR ((nutrition* OR diet*) N2 (therap* OR advice OR intervention# OR treatment#)) OR (education* N2 (care OR intervention* OR therap* OR patient# OR material)) OR ("nurse led" N2 (care OR intervention* OR therap*)) OR (social N2 intervention*) OR ("patient care" N2 continuity) OR (care N2 continuum) OR (patient# N2 discharg*) OR (patient# N2 (handoff# OR "hand over")) OR ((patient# OR care) N2 (transfer# OR transition#)) OR (care N2 episode#))) OR AB(("supportive care" OR rehabilitat* OR "re-habilitat*" OR prerehabilitat* OR "pre-rehabilitat*" OR prehabilitat* OR "pre-habilitat*" OR "allied health care" OR "physical therap*" OR physiotherap* OR "exercise therap*" OR exercise OR physiotherap* OR "physical therap*" OR kinesiotherap* OR aftercare OR "after care" OR "after treatment#" OR "follow-up care#" OR "social work" OR "psychosocial support" OR "psychosocial care" OR counseling OR psychotherap* OR psychologist# OR "return to work" OR "back to work" OR "occupational therap*" OR ergotherap* OR "long term care") OR ((exercise# N2 (therap* OR remedial)) OR ((nutrition* OR diet*) N2 (therap* OR advice OR intervention# OR treatment#)) OR (education* N2 (care OR intervention* OR therap* OR patient# OR material)) OR ("nurse led" N2 (care OR intervention* OR therap*)) OR (social N2 intervention*) OR ("patient care" N2 continuity) OR (care N2 continuum) OR (patient# N2 discharg*) OR (patient# N2 (handoff# OR "hand over")) OR ((patient# OR care) N2 (transfer# OR transition#)) OR (care N2 episode#))) OR DE(("supportive care" OR rehabilitat* OR "re-habilitat*" OR prerehabilitat* OR "pre-rehabilitat*" OR prehabilitat* OR "pre-habilitat*" OR "allied health care" OR "physical therap*" OR physiotherap* OR "exercise therap*" OR exercise OR physiotherap* OR "physical therap*" OR kinesiotherap* OR aftercare OR "after care" OR "after treatment#" OR "follow-up care#" OR "social work" OR "psychosocial support" OR "psychosocial care" OR counseling OR psychotherap* OR psychologist# OR "return to work" OR "back to work" OR "occupational therap*" OR ergotherap* OR "long term care") OR ((exercise# N2 (therap* OR remedial)) OR ((nutrition* OR diet*) N2 (therap* OR advice OR intervention# OR treatment#)) OR (education* N2 (care OR intervention* OR therap* OR patient# OR material)) OR ("nurse led" N2 (care OR intervention* OR therap*)) OR (social N2 intervention*) OR ("patient care" N2 continuity) OR (care N2 continuum) OR (patient# N2 discharg*) OR (patient# N2 (handoff# OR "hand over")) OR ((patient# OR care) N2 (transfer# OR transition#)) OR (care N2 episode#))) )
