# Supplementary material for: Socioeconomic position and access to supportive cancer care in Europe: a scoping review of literature from 2000 to 2024
Source: J Cancer Surviv. 2025 Oct 7;20(5):2649–67. doi: 10.1007/s11764-025-01904-3 (PMC13723617; doi:10.1007/s11764-025-01904-3)
Supplement: Supplementary file 2 [file 11764_2025_1904_MOESM2_ESM.docx]

| **Author**  **Year**  **Country** | **Relationship** to  **Supportive care type** | **Study design (N)**  **Data source**  **SEP measure**  **Statistical method** | **Information on adjustment** | **Adjusted OR/HR**  *Proportions are reported in absence of OR/HR* | | | | **Finding (between SEP and access/uptake)**  **Statistically significant indicated with an asterisk* |
| --- | --- | --- | --- | --- | --- | --- | --- | --- |
|  |  |  |  | **Education*** | | **Income*** | |  |
| Hensel, M.    2002  Germany | **Uptake** of **rehabilitation services** (according to patient needs or not specified) | Cross-sectional study  (304)  Questionnaire; Clinical records  Education  Descriptive statistics | NA | Intergroup comparison:  **Rehabilitation users** (n=139) Elementary school: 98 (70.5%)  High school or higher: 39 (28.1%)  **Non-users of rehabilitation** (n=165)  Elementary school: 103 (62.4%)  High school or higher: 54 (32.7%) | |  | | No association  Not significant |
| Waldmann, A.  2007  Germany | **Uptake** of **rehabilitation services** (according to patient needs or not specified) | Historical cohort study with retrospective data collection (241)  Registry data; questionnaire  Education  Multivariate analysis | Adjusted for personal, clinical and therapeutic variables | **Rehabilitation use:**  **Ref**: <9 years;  **OR** 9-12 years: **1.2** [0.6 -2.4];  **OR** >12 years : **0.9** [0.3 – 2.6] | |  | | No significant influence of education on the use of rehabilitation services could be found.  Not significant |
| Geyer, S.  2012  Germany | **Uptake** of **rehabilitation services** (according to patient needs or not specified) | Prospective cohort study (238)  Interview data  Education  Logistic regression analysis | Adjusted for (at least): the number of children under 12 in the household, tumor stage, type of follow up treatment. | **Ref**: no degree/main school;  **OR** secondary school: 0.62 [0.30 -1.30];  **OR** university entrance qualification: **0.37** [0.14 – 0.96] | | - | | The probability of using oncological rehabilitation decreased with increased education levels.  Not significant |
| Nicolaije, K.A.H.  2012  The Netherlands | **Access** to **information about supportive care** | Cross-sectional study  (742)  Questionnaire (EORTC-QLQ INFO25);  Cohort registry (PROFILES)  Education  Multivariate linear and binary regression analysis | Adjusted for: sociodemographic and clinical characteristics | **β:**  Ref: High education; Intermediate education:  -0.07;  Low education:  -0.02 | |  | | High SEP was associated with higher information receipt  Not significant |
| **Author**  **Year**  **Country** | **Relationship** to  **Supportive care type** | **Study design (n)**  **Data source**  **SEP measure**  **Statistical method** | **Information on adjustment** | **Adjusted OR/HR**  *Proportions are reported in absence of OR/HR* | | | | **Finding (between SEP and access/uptake)**  **Statistically significant indicated with an asterisk* |
|  |  |  |  | **Education*** | | **Income*** | |  |
| von Heymann-Horan, A.B.  2012  Denmark | **Uptake** of **psychosocial services** | Prospective cohort study (3646)  Questionnaire; national registry  Education  Multivariate cox regression models | Adjusted for age at diagnosis, gender, education, previous psychotherapy use, emotional support from partner, children, and other family members, with SF-36 mental and physical component summaries, Charlson index scores | **Ref**: <8 years;  **HR** 8-10 years: **1.23** [0.69, 2.17];  **HR** >10 years: **1.97*** [1.06,3.65] | |  | | Longer education (over 10 years compared to less than 8) was associated with greater likelihood of initiating psychotherapy *  p-value <0.05 |
| Holm, L.V.  2013  Denmark | **Uptake** of **psychosocial services**;  **Physical rehabilitation services** | Population based cohort study (3439)  Questionnaire; National registry; Clinical records  Education;  Income  Univariate and multiple logistic regression analyses | Adjusted for: age group and cancer diagnosis | **MEN**  **Physical care**:  **Ref:** <10 years; **OR** 10-12 years: **1.11** [0.85-1.45]; **OR** > 12 years: **1.22** [0.88-1.68]  **Psychological care:**  **Ref**: <10years;  **OR** 10-12 years: **0.96** [0.62-1.48]; **OR** >12 years: **1.65*** [1.02-2.67] | **WOMEN**  **Physical care**: **Ref** <10 years; **OR** 10-12 years: **1.38** [01.10-1.74];  **OR** > 12 years: **2.26**** [1.73-2.96]  **Psychological care:**  **Ref** <10years; **OR** 10-12 years: **1.31** [0.98-1.75];  **OR** >12 years: **2.17**** [1.61-2.93] | **MEN**  **Physical care:** **Ref** low;  **OR** medium: **1.67**** [1.21-2.30];  **OR** high: **1.55*** [1.07-2.24]  **Psychological care:**  **Ref** low;  **OR** medium:  **1.15** [0.69-1.93];  **OR** high: **1.14** [0.64-2.04] | **WOMEN**  **Physical care:** **Ref** low;  **OR** medium: **1.40**** [1.09-1.79];  **OR** high: **2.07**** [1.52-2.80]  **Psychological care:**  **Ref** low;  **OR** medium: **1.30** [0.94-1.80];  **OR** high: **1.41** [0.98-2.05] | - Women with a higher education and to some extent higher income had increased odds of participating in activities* - Men with a higher income had increased odds of participating in physical activities* - A high education was associated with participation in psychological activities for both men and women*   p-value: < 0.001  (both income and education) |
| **Author**  **Year**  **Country** | **Relationship** to  **Supportive care type** | **Study design (n)**  **Data source**  **SEP measure**  **Statistical method** | **Information on adjustment** | **Adjusted OR/HR**  *Proportions are reported in absence of OR/HR* | | | | **Finding (between SEP and access/uptake)**  **Statistically significant indicated with an asterisk* |
| Zeissig, S.R.  2015  Germany | **Uptake** of **psychosocial services** | Historical cohort study with retrospective data collection (6143)  Education;  Income  Multivariate logistic regression analysis | Adjusted for: Gender, age, place of residence, education, income, and tumor spread at diagnosis | **Psycho-oncological services:**  **Ref**: <10years;  **OR** 10+ years: **1.5** [1.2 -1.8];  **OR** unknown: **0.9** [0.3 – 2.3]  **Cancer counselling centers:**  **Ref**: <10years;  **OR** 10+ years: **2.1** [1.5 -3.0];  **OR** unknown: **2.0** [0.5 – 9.0] | | **Psycho-oncological services:**  **Ref:** Above the poverty line  **OR** poor: **1.1** [0.3-1.5]  **OR** unknown: 0.8 [0.8-1.1]  **Cancer counselling centers:**  **Ref:** Above the poverty line  **OR** poor: 1.0 [0.5-1.7]  **OR** unknown: 1.0 [0.3-1.2] | | A higher level of education was significantly associated with the use of cancer counselling and the use of psycho-oncological services*, but income level was not.  p-value <0.01 |
| Ture, M.  2015  Switzerland | **Uptake** of **rehabilitation services** **(according to patient needs or not specified)** | Cross-sectional study (238)  Questionnaire; Clinical records  Education  Univariate analysis | No multivariable adjustment | **MEN**  **Rehabilitation users** (n=58):  Obligatory school: 1 (1.7%)  Apprenticeship: 31 (53.4%)  High school:12 (20.7%)  University: 14 (24.1%)  **Rehabilitation nonusers** (n=65):  Obligatory school: 7 (10.8%)  Apprenticeship: 29 (44.6%)  High school:6 (9.2%)  University: 23 (35.4%) | **WOMEN**  **Rehabilitation users** (n=43):  Obligatory school: 9 (20.9%)  Apprenticeship: 19 (44.2%)  High school: 6 (14.0%)  University: 9 (20.9%)  **Rehabilitation nonusers** (n=72):  Obligatory school: 7 (9.9%)  Apprenticeship: 38 (53.9%)  High school: 10 (14.1%)  University: 16 (22.5%) | - | - | Stratified analyses showed that male patients with lower education were more likely to use rehabilitation.*  p-value=0.039 (men)  p-value=0.417 (women) |
| Moutsten, I.R.  2015  Denmark | **Access** to **rehabilitation services (according to patient needs)** | Population based cohort study (13840)  National registry; Danish Cancer Registry  Education;  Income  Multivariate analysis | Adjusted for: sex, age, diagnosis, disposable income, cohabitation status and no. of children living at home | **Ref**: short education;  **HR** medium education: **1.14** [1.02-1.27];  **HR** long education: **1.26** [1.12-1.43];  **HR** unknown: **0.51** [0.39-0.66] | | - | | Association between high education and rate of referral to municipality rehabilitation*  Significant but p-value undisclosed |
| Arts, L.P.J.  2018  The Netherlands | **Uptake** of **psychosocial** **services** | Cross-sectional study (1444)  Questionnaire; Cohort registry (PROFILES)  Education  Multivariate logistic regression analysis | Adjusted for: age, sex  comorbidities | **Ref**: middle education;  **OR** low education: **1.32** [0.93-1.88];  **OR** high education: **1.02** [0.70-1.47] | | - | | Low education level was associated with increased use of psychosocial care services  Not significant |
| **Author**  **Year**  **Country** | **Relationship** to  **Supportive care type** | **Study design (n)**  **Data source**  **SEP measure**  **Statistical method** | **Information on adjustment** | **Adjusted OR/HR**  *Proportions are reported in absence of OR/HR* | | | | **Finding (between SEP and access/uptake)**  **Statistically significant indicated with an asterisk* |
|  |  |  |  | **Education*** | | **Income*** | |  |
| Ernst, J.  2018  Germany | **Access** to **psychosocial services** | Cross-sectional study (4020)  Questionnaire  Education  Binary regression analysis | Adjusted for: Sociodemographic, psychosocial, disease related factors | **OR** higher level education: **1.25** [1.04-1.51] | |  | | Higher education had a strong effect on recommendation/referral to psychosocial support services*  Significant but p-value undisclosed |
| Garmy, P.  2018  Sweden | **Uptake** of **psychosocial services;**  **physical rehabilitation services** | Cross-sectional study (168)  Questionnaire  Education  Descriptive statistics | No multivariable adjustment | Intergroup comparison:  **Users** (n=95, 56.5%):  Compulsory school: 17 (18.1%)  Secondary school: 41 (43.6%)  College/university: 36 (38.3%)  **Nonusers** (n=73, 43.5%)  Compulsory school: 21 (28.8%)  Secondary school: 29 (39.7%)  College/university: 23 (31.5%) | | - | | No association  p-value: 0.253 |
| Hofreuter-Gätgens, K.  2018  Germany | **Uptake** of **psychosocial services; physical rehabilitation services** | Prospective cohort study  (455)  Questionnaires  Education;  Income  Logistic regression analysis | Adjusted for: Age, disease severity, psychological comorbidities | **Breast cancer patients:**  **Psychosocial measures:**  **Ref** low-medium level of education;  **OR** high level of education: **2.30** [1.21-4.40]*  **Medical rehabilitation:**  **Ref** low-medium level of education;  **OR** high level of education: **1.05** [0.54-2.01] | **Prostate cancer patients:**  **Psychosocial measures:**  **Ref** low-medium level of education;  **OR** high level of education: **1.05** [0.48-2.31]  **Medical rehabilitation:**  **Ref** low-medium level of education;  **OR** high level of education: **0.89** [0.50-1.58] | **Breast cancer patients:**  **Psychosocial measures:**  **Ref** net equivalent income;  **OR** high net equivalent income: **1.60** [0.76-3.33]  **Medical rehabilitation:**  **Ref** net equivalent income;  **OR** high net equivalent income: **1.61** [0.81-3.18] | **Prostate cancer patients:**  **Psychosocial measures:**  **Ref** net equivalent income;  **OR** high net equivalent income: **0.54** [0.23-1.31]  **Medical rehabilitation:**  **Ref** net equivalent income;  **OR** high net equivalent income: **0.83** [0.46-1.52] | Only in breast cancer patients did education increase the likelihood of participating in psychosocial rehabilitation.*  p-value ≤ 0.05 |
| Weis, J.  2018  Germany | **Uptake** of **psychosocial services** | Cross-sectional study (1398)  Questionnaire; Clinical records  Education  Multivariate logistic regression analysis | Adjusted for: age,  Sex, cancer type | **Ref:** high level (over 10 y);  **OR** no/other education: **0.862** [0.353-2.105];  **OR** secondary school education **(5-9 y):** **0.768** [0.564-1.047];  **OR** secondary school education **(10 y): 0.815** [0.605-1.099] | |  | | Higher education level was associated with the use of psychological care  Not significant |
| **Author**  **Year**  **Country** | **Relationship** to  **Supportive care type** | **Study design (n)**  **Data source**  **SEP measure**  **Statistical method** | **Information on adjustment** | **Adjusted OR/HR**  *Proportions are reported in absence of OR/HR* | | | | **Finding (between SEP and access/uptake)**  **Statistically significant indicated with an asterisk* |
|  |  |  |  | **Education*** | | **Income*** | |  |
| Dalton, S.O.  2019  Denmark | **Access** to; **Uptake** of **rehabilitation services** (according to patient needs) | Population based cohort study (13059)  National registry; Danish cancer registry  Education;  Income  Multivariate cox regression models | HRs (education) adjusted for: age, year of diagnosis  HRs (income) adjusted for: age, year of diagnosis, education | **MEN**  **Referral (Access):**  **Ref** short education;  **HR** medium education: **1.2**3 [1.02-1.49];  **HR** long education: **1.30** [ 1.06–1.59]  **Attendance (Uptake):**  **Ref** short education;  **HR** medium education: **1.32** [1.04-1.68];  **HR** long education: **1.72** [1.35–2.20] | **WOMEN**  **Referral (Access):**  **Ref** short education;  **HR** medium education: **1.07** [0.92-1.23];  **HR** long education: **1.32** [1.15–1.53]  **Attendance (Uptake):**  **Ref** short education;  **HR** medium education: **1.17** [0.99-1.38];  **HR** long education: **1.56** [1.33–1.84] | **MEN**  **Referral (Access):**  **Ref** Q1;  **HR** Q2: **0.93** [0.76-1.13];  **HR** Q3 **0.92** [0.75-1.14];  **HR** Q4: **0.98** [0.79-1.21];  **HR** Q5 **0.95** [0.78-1.16]  **Attendance (Uptake):**  **Ref** Q1;  **HR** Q2: **1.15** [0.92-1.45];  **HR** Q3: **1.24** [0.98-1.57];  **HR** Q4: **1.06** [0.83-1.35];  **HR** Q5: **1.09** [0.87-1.37] | **WOMEN**  **Referral (Access):**  **Ref** Q1;  **HR** Q2: **0.92** [0.78-1.08];  **HR** Q3: **1.01** [0.86-1.19];  **HR** Q4: **1.01** [0.86-1.18];  **HR** Q5: **1.03** [0.88-1.21]  **Attendance (Uptake):**  **Ref** Q1;  **HR** Q2: **1.18** [0.98-1.41];  **HR** Q3: **1.16** [0.97-1.39];  **HR** Q4: **1.23** [1.03-1.47];  **HR** Q5: **1.03** [1.03-1.46] | Compared to individuals with short education, men and women with long education and men with medium education had higher adjusted hazard ratios (HR) for being referred to rehabilitation (**access**). Long education was associated with attendance (**uptake**) in both genders while in men, medium education and in women high income, respectively was associated with attendance (**uptake**).*  Significant but p-value undisclosed |
| Schuurhuizen, C.S.E.W.  2019  The Netherlands | **Uptake** of **psychosocial services** | Secondary analysis of trial data (349)  Data from TES trial (NTR4034)  Education  Two-step multivariate analysis | Adjusted for: need to talk to professional, pain score at baseline, age, education (included in the multivariable model) | **Ref** low education;  **OR** middle education: **1.255** [0.270-5.837];  **OR** high education: **2.518** [0.527-12.024] | | - | | Patients who completed higher education levels were found to be more likely to use psychosocial services.*  p-value: 0.041 |
| Breidenbach, C.  2020  Germany | **Uptake** of **psychosocial services** | Prospective cohort study (3094)  Questionnaire; TrueNTH Global registry  Education  Multilevel analysis | Adjusted for**:** individual patient and structural center characteristics  Adjusted for **mediator**: treatment | Intergroup comparison:  **Users** (n=1134):  Lower secondary school: 474 (41.8%)  Intermediate secondary school: 264 (23.3%)  Entrance certificate for university: 331 (29.2%)  **Nonusers** (n=1960)  Lower secondary school: 712 (36.3%)  Intermediate secondary school: 466 (23.8%)  Entrance certificate for university: 644 (32.9%) | | - | | No significant association between education and use of psychosocial services |
| Osowiecka, K.  2020  Poland | **Uptake** of **psychosocial services** | Cross-sectional study (384)  (Orally administered) questionnaire  Education  Descriptive statistics; Chi-square test | - | Intergroup comparison:  **Users** (n=290):  Primary school: 53 (18%)  Secondary school: 171 (59%)  High school: 59 (20.5%)  No data: 7 (2.5%)  **Nonusers** (n=29):  Primary school: 7 (24%)  Secondary school: 17 (59%)  High school: 5 (17%)  No data: 0 | |  | | No significant association between education level and psychological support or supportive care was found  between users, non-users, and those who do not know or need psychological support.  p-value: 0.91 |
| **Author**  **Year**  **Country** | **Relationship** to  **Supportive care type** | **Study design (n)**  **Data source**  **SEP measure**  **Statistical method** | **Information on adjustment** | **Adjusted OR/HR**  *Proportions are reported in absence of OR/HR* | | | | **Finding (between SEP and access/uptake)**  **Statistically significant indicated with an asterisk* |
|  |  |  |  | **Education*** | | **Income*** | |  |
| Ousseine, Y.M.  2020  France | **Access** to **psychosocial services** | Cross-sectional study (4045)  (Orally administered) Questionnaire; FNHI registry  Education; Income  Multivariate analysis | Adjusted for: sociodemographic, medical, and psychosocial characteristics | - | | **Ref** high income;  **OR** low income **3.62** [2.57-5.11]; **OR** medium income: **2.06** [1.49-2.83];  **OR** unknown: **1.54** [0.92-2.59] | | Contact with a social worker increased with a low income*  p-value: 0.007 |
| Breidenbach, C.  2021  Germany  Switzerland | **Uptake** of **psychosocial services** | Prospective cohort study (19865)  Questionnaire; TrueNTH Global Registry  Education  Logistic multilevel analysis | Adjusted for:  sociodemographic and clinical patient information, therapy information | **Ref** lower secondary school  **OR** no qualification**: 0.59** [0.30-1.17];  **OR** intermediate secondary school**: 1.05** [0.92-1.20];  **OR** vocational technical diploma**: 1.11** [0.94-1.30];  **OR** entrance certificate university**: 0.87** [0.76-0.99];  **OR** other qualification**: 1.11** [0.74-1.65] | |  | | Patients with an entrance certificate for university have a lower likelihood to use SSC than patients with lower secondary school education.*  p-value: 0.04 |
| Noeres, D.  2021  Germany | **Uptake** of **rehabilitation services (according to patient needs)** | Prospective cohort study (409)  Questionnaires  Education  Multivariate analysis | Adjusted for: physical and mental health scores (SF-12) | **Ref** Basic secondary school leaving certificate  **OR** Intermediate school certificate**: 4.23** [1.16-11.38];  **OR** University entrance qualification**: 3.62** [1.28-5.90] | | - | | Higher education had a significant effect on the likelihood of participating in rehabilitation.*  p-value: 0.01 (Intermediate school certificate)  p-value: 0.03 (University entrance qualification) |
| Orange, S.T.  2021  United Kingdom | **Access** to **information as supportive care** | Cross-sectional study (242)  Questionnaire  Education  Univariate generalized linear model | Adjustment for **confounders**: demographic characteristics | **Ref** other  **OR** academic degree or professional qualification**: 1.0** [0.62-1.8] | | - | | There was no evidence of associations between sociodemographic characteristics and the recall of physical activity advice  Not significant |
| **Author**  **Year**  **Country** | **Relationship** to  **Supportive care type** | **Study design (n)**  **Data source**  **SEP measure**  **Statistical method** | **Information on adjustment** | **Adjusted OR/HR**  *Proportions are reported in absence of OR/HR* | | | | **Finding (between SEP and access/uptake)**  **Statistically significant indicated with an asterisk* |
|  |  |  |  | **Education*** | | **Income*** | |  |
| Scherer-Trame, S.  2021  Germany | **Uptake** of **rehabilitation services** (not specified) | Retrospective cohort study (3704)  DACHS Study data; National registry; Clinical records  Education  Multivariate logistic regression analysis | Adjustment for confounders: age, sex, partnership, education, employ- ment status, type of health insurance, BMI, smoking, physical activity level, cancer stage, tumor location, Charlson Comorbidity Score, chemotherapy, radio- therapy, surgical technique and surgical volume of treating hospital | Intergroup comparison:  **Users** (n=1615)  Less than 9 years of education: 1042 (43.5%)  10 years of education: 303 (45.7%)  More than 10 years of education:  266 (41.5%)  **Nonusers** (n=2089)  Less than 9 years of education: 1351 (56.5%)  10 years of education: 360 (54.3%)  More than 10 years of education:  375 (58.5%) | |  | | There were no significant (inverse) associations between years of school education and IRT use  p-value: 0.3096 |
| Korn, P.    2022  Germany | **Uptake** of **psychosocial services; physical rehabilitation services** | Retrospective cohort study (1532)  Questionnaire; Cohort registry (DÖSAK)  Education  Intergroup comparison | - | Intergroup comparison:  **Users** (n=542)  None or low: 18 (3.3%)  Middle: 410 (75.6%)  High: 111 (20.5%)  **Nonusers** (n=990)  None or low: 27 (2.7%)  Middle: 745 (75.6%)  High: 206 (20.8%) | |  |  | Education levels did not seem to be decisive in determining rehabilitation participation, difference was not statistically significant  p-value: 0.067 |
| **Author**  **Year**  **Country** | **Relationship** to  **Supportive care type** | **Study design (n)**  **Data source**  **SEP measure**  **Statistical method** | **Information on adjustment** | **Adjusted OR/HR**  *Proportions are reported in absence of OR/HR* | | | | **Finding (between SEP and access/uptake)**  **Statistically significant indicated with an asterisk* |
|  |  |  |  | **Education*** | | **Income*** | |  |
| Singer, S.  2022  Germany | **Uptake** of; **Access** to **psychosocial services**  Purpose: **Exploratory** | Prospective cohort study (456)  Questionnaire; Clinical records  Education;  Income | Adjusted for: all other variables in the model (age, migration status, risk, financial issues) | **Ref** <10 years  **SOCIAL SERVICES ACCESS**: **OR** >10 years**: 1.2** [0.9-1.6]  **SOCIAL SERVICES UPTAKE:** **OR** >10 years: **1.2** [0.9-1.6]  **Ref** <10 years  **PSYCHOLOGICAL SERVICES ACCESS: OR** >10 years**: 1.0** [0.8-1.4]  **PSYCHOLOGICAL SERVICES UPTAKE**: **OR** >10 years: **1.0** [0.8-1.4] | | **Ref** high income  **SOCIAL SERVICES ACCESS**: **OR** middle income**: 1.3** [0.7-2.3]; **OR** low income**: 1.1 [**0.6-2.0]  **SOCIAL SERVICES UPTAKE:** **OR** middle income: **1.0** [0.6-1.8], **OR** low income: **1.0** [0.5-1.9]  **Ref** high income  **PSYCHOLOGICAL SERVICES ACCESS:** **OR** middle income: **1.0** [0.6-1.8], **OR** low income: **0.6** [0.3-1.1]  **PSYCHOLOGICAL SERVICES UPTAKE**: **OR** middle income: **1.0** [0.6-1.7], **OR** low income OR: **1.4** [0.7-2.6] | | Education and income were not statistically significant predictors of offer or use for both social services and psychological services.  Not significant |
| Cogollos‐de‐la‐Peña, R.  2023  Spain | **Uptake** of **physical rehabilitation services** | Cross-sectional cohort study (2235)  Questionnaire; Cohort registry (DAMA)  Education  Multivariate Poisson regression model | No information provided | **Visiting** (386):  None or primary: 39 (10.1%)  Secondary: 137 (35.5%)  Superiors: 210 (54.4%)  **Non-visiting** (1699):  None or primary: 395 (23.2%)  Secondary: 627 (36.9%)  Superiors: 677 (39.9%) | | - | | Women with a higher level of education had increased access to physiotherapy.*  p-value: <0.001 |
| Ekels, A.  2024  The Netherlands | **Uptake** of **psychosocial services**;  **Physical rehabilitation services** | Retrospective cohort study (2319)  Questionnaire; Cohort registry (PROFILES)  Income;  Education  Binary logistic regression | No information provided | **Ref** low education  **PSYCHOSOCIAL CARE UPTAKE:**  **OR** high education**: 1.5** (p=0.04)  **Ref** low education  **PHYSICAL CARE UPTAKE:**  **OR** high education**: 1.7** (p=0.01) | | **Ref** low income level  **PSYCHOSOCIAL CARE UPTAKE:**  **OR** high income level**: 1.5** (p=0.03) | | Patients with a high educational level reported statistically significantly higher use of additional physical and psychosocial care during the first 2 years after diagnosis, compared to patients with a low educational level. Patients with a high income level reported significantly higher utilization of psychosocial care from 2 years after diagnosis, compared to those with a low income level.*  p-value: 0.04 (education and psychosocial care)  p-value: 0.01 (education and physical care)  p-value: 0.03 (income and psychosocial care) |
| **Author**  **Year**  **Country** | **Relationship** to  **Supportive care type** | **Study design (n)**  **Data source**  **SEP measure**  **Statistical method** | **Information on adjustment** | **Adjusted OR/HR**  *Proportions are reported in absence of OR/HR* | | | | **Finding (between SEP and access/uptake)**  **Statistically significant indicated with an asterisk* |
|  |  |  |  | **Education*** | | **Income*** | |  |
| Strömberg, U.  2024  Sweden | **Access** to **physical rehabilitation services** | Population based cohort study (6505)  National registry data (PCBaSe; NPCR)  Education;  Income  Multivariate cox regression models | Adjusted for: age, T stage, Gleason score, number of positive biopsy scores, prostate-specific antigen value, healthcare region at the time of diagnosis, individual/neighborhood-level socioeconomic variables | **Ref** high education  **OR** intermediate education**: 0.99** [0.87-1.13];  **OR** low education**: 0.82** [0.69-0.97] | | **Ref** high income  **OR** intermediate income**: 1.02** [0.90-1.15];  **OR** low income**: 0.90** [0.74-1.10] | | A statistically significant association for individual-level education (low versus high academic education) and rehabilitation was found.*  Significant but p-value undisclosed |
| Ziegler, E.  2024  Germany | **Access** to **psychosocial services** | Cross-sectional study (1121)  Questionnaire  Education  Binary Logistic regression | Adjusted for: Age, sex, primary cancer type, years since diagnosis, decision making preference, health insurance, central contact person available | **Ref** low education  **OR** medium education**: 1.11** [0.67-1.85];  **OR** high education**: 1.02** [0.63-1.65] | | - | | No significant associations between education and provision of psycho-oncological counselling were found.  p-value: 0.686 (medium education)  p-value: 0.942 (high education) |

***Appendix A*.** *Complete overview of the included studies.*

**Data for education or income shown for sex combined, unless indicated otherwise.*

***Statistical significance is indicated as defined by the original study.*
